# Electrophysiological characterization of a diverse group of sugar transporters from *Trichoderma reesei*

**DOI:** 10.1038/s41598-021-93552-7

**Published:** 2021-07-19

**Authors:** Sami Havukainen, Jonai Pujol-Giménez, Mari Valkonen, Ann Westerholm-Parvinen, Matthias A. Hediger, Christopher P. Landowski

**Affiliations:** 1 Protein Production Team, VTT Technical Research Center of Finland Ltd, Tietotie 2, 02150 Espoo, Finland; 2 Membrane Transport Discovery Lab, Department of Biomedical Research, Inselspital, University of Bern, 3010 Bern, Switzerland

**Keywords:** Monosaccharides, Polysaccharides, Membrane proteins, Voltage clamp, Industrial microbiology, Fungal biology, Fungal physiology, Fungal genes, Saccharomyces cerevisiae, Xenopus, Genetic engineering

## Abstract

*Trichoderma reesei* is an ascomycete fungus known for its capability to secrete high amounts of extracellular cellulose- and hemicellulose-degrading enzymes. These enzymes are utilized in the production of second-generation biofuels and *T. reesei* is a well-established host for their production. Although this species has gained considerable interest in the scientific literature, the sugar transportome of *T. reesei* remains poorly characterized. Better understanding of the proteins involved in the transport of different sugars could be utilized for engineering better enzyme production strains. In this study we aimed to shed light on this matter by characterizing multiple *T. reesei* transporters capable of transporting various types of sugars. We used phylogenetics to select transporters for expression in *Xenopus laevis* oocytes to screen for transport activities. Of the 18 tested transporters, 8 were found to be functional in oocytes. 10 transporters in total were investigated in oocytes and in yeast, and for 3 of them no transport function had been described in literature. This comprehensive analysis provides a large body of new knowledge about *T. reesei* sugar transporters, and further establishes *X. laevis* oocytes as a valuable tool for studying fungal sugar transporters.

## Introduction

Lignocellulose biomass has gained interest as a feedstock for second generation biofuels due to its high abundance and renewability. It is used as a nutrition source by saprophytic fungi, which degrade its polysaccharide components into a range of soluble sugars. *Trichoderma reesei* is a well-known species of saprophytic fungi that is used in the enzyme industry for the production of cellulose- and hemicellulose degrading enzymes (cellulases and hemicellulases), and which has been also studied as a platform for heterologous protein production. Although *T. reesei* has been the subject of numerous publications since its identification in the 1950s, sugar transporters coded in its genome remain poorly characterized.

Sugar transporters are membrane proteins which translocate sugars across the cell membrane. Fungal sugar transporters belong mainly to the major facilitator superfamily (MFS)^1^, whose members are characterized by the 12 transmembrane domain (TMD) topology^2^. Few 7 TMD SWEETs (**S**ugars **W**ill **E**ventually be **E**xported **T**ransporter) have also been identified from fungi, but they are absent from phylum Ascomycota which contains industrially important fungi such as *T. reesei* and *S. cerevisiae*^3^. The MFS contains both passive facilitators and sugar/H^+^ symporters. Passive facilitators transport sugars along their concentration gradient across the cell membrane, while symporters are able to accumulate sugars against their concentration gradient by coupling the transport process to the transport of a co-substrate which follows its concentration gradient. In the known fungal sugar symporters the co-substrates are protons, in contrast to sodium ions used by many sugar symporters from animals, that belong to a different family of transporters^4^.

The yeast *Saccharomyces cerevisiae* is the most studied species of fungi, and relevant to biofuel processes as the organism responsible for fermenting the lignocellulose-derived sugars to ethanol. Its well-characterized sugar transport system relies solely on passive facilitators in the transport of hexose sugars, which are present at high concentrations in its natural habitat^5^. On the other hand, the natural habitat of saprophytic fungi contains wide variety of biomass-derived sugars which are available only at low concentrations^6^. This wider range of utilizable substrates is reflected by the high number of sugar transporter genes when compared to *S. cerevisiae*^7^. The scarcity of sugars in the natural habitat is reflected by the fact that fungal high-affinity monosaccharide transporters often have their affinities in the micromolar range^7–11^, whereas the affinities of their *S. cerevisiae* counterparts are in the millimolar range^12^. Additionally, many fungal monosaccharide transporters appear to utilize the active sugar/H^+^ symport mechanism, again unlike *S. cerevisiae*, which further suggests adaptation to low sugar environments^10^. Fungal sugar transporters have been proven useful for engineering *S. cerevisiae* to uptake biomass-derived sugars (e.g. d-xylose, l-arabinose and d-galacturonic acid) whose transport is not otherwise optimal in yeast, and whose utilization is required for an economically competitive bioethanol production process^13–15^.

Although the genome of *T. reesei* has been predicted to contain about 50–100 genes coding for sugar transporters^1,7,16^, only handful of them have been characterized in the literature (Reviewed in Ref.^1^). Transporters have thus far been identified for monosaccharides, $\upbeta$-linked disaccharides and sugar acids^7,14,17–21^. Several transporter genes have been observed to be upregulated when the fungus is grown on cellulase-inducing carbon sources, and to be regulated by transcription factors involved in cellulase induction^22–25^. As different sugars act as inducers or repressors of the cellulolytic system and since these sugars are believed to be sensed intracellularly, sugar transporters play an important role in cellulase induction in saprophytic fungi^26,27^. Indeed, the deletion of sugar transporter genes has been shown to affect cellulase production, sugar transport and growth in *T. reesei*^6,17,19,23,28^.

Inducers of the cellulase and hemicellulase gene expression in *T. reesei* include cellobiose, sophorose, lactose and l-sorbose^26^. Of these, lactose is relevant to industrial applications due to its low price and solubility^23^. d-Glucose, which is easily metabolized, represses the production of cellulases and hemicellulases via phenomenon termed carbon catabolite repression (CCR)^29^. The preference for d-glucose is illustrated by the fact that many fungal species possess multiple glucose transporters with varying affinities, capacities and expression patterns^8,12^. The tight regulation of cellulase induction by different sugars could possibly enable the improvement of cellulase production via manipulation of sugar transporters. This would be desirable, as these enzymes form a significant fraction of the manufacturing cost of second generation bioethanol^30^. Examples would be the alleviation of CCR by deletion of d-glucose transporters, or the improvement of inducer uptake by overexpression of e.g. lactose transporters. The prerequisite for both of these approaches is the knowledge of the sugar transportome of the species in question.

Fungal sugar transporters have been characterized mainly by heterologous expression in *S. cerevisiae* with transport experiments carried out with radiolabeled sugars. Transporters from other kingdoms of life have been characterized with electrophysiological methods (Reviewed for plant transporters in Ref.^31^), such as two-voltage electrode clamp (TEVC)^32^, but to our knowledge there are only two published studies where this method has been applied to fungal sugar transporters^33,34^. In this method, the transporter gene is expressed in *Xenopus laevis* oocytes and the flow of ions through their plasma membrane is monitored^32^. To do so, the cell membrane is clamped to a specific voltage (analogous to physiological resting membrane potential) and the current required to keep this voltage constant is recorded. Electrophysiological studies are convenient for transporter functional characterization, since they do not require expensive and limitedly available radiolabeled sugars, and since a complete set of kinetics can be measured on a single oocyte simultaneously with multiple test voltages^35^. Nevertheless, since these methods measure the flow of ions into the cell, they are limited to the characterization of electrogenic transporters, e.g. sugar/H^+^ symporters. In this regard, many fungal sugar transporters appear to function as symporters, and thus they could be studied with this method.

Accordingly, we set to investigate *T. reesei* transporters with electrophysiological methods. Our aim was to gain better understanding of *T. reesei* sugar transporters, and to further establish the TEVC method for the analysis of fungal sugar transporters. We used phylogenetic analysis to identify a convenient set of transporters for the functional studies, with the aim of selecting transporters for many different classes of sugars. Some of these transporters had been published before, but our analysis provided new information about their substrates and kinetics. We also identified and characterized some transporters for which no transport activity had been described before.

**Table 1:** *T. reesei* transporters tested in this study for symport activity in oocytes and their regulation by carbon sources and transcription factors.

Trire2	Clade	C-source		Transcription factor			H^+^	Substrates		Refs.
		Cel/Lac	Lac	XYR1	ACE3	CRE1		New	Known	
50618	Mono/c6	$\uparrow$				$\uparrow$	x	fru		
72383	Mono/c6									
79202^a^	Mono/c6	$\uparrow$	$\uparrow$		$\downarrow$	$\uparrow$				
106556	Mono/c6	$\downarrow$	$\downarrow$			${\downarrow }$				
STR3^a^	Mono/c6						x	sor	glc, fru, gal, mann, xyl	^7^
62502	Mono/c5	$\uparrow$								
STR1	Mono/c5	$\uparrow$	$\uparrow$	$\downarrow$			x	ara	glc, fru, gal, mann, xyl	^7,17^
56684^a^	Di/$\upbeta$	$\uparrow$	$\uparrow$		$\downarrow$					
67752^a^	Di/$\upbeta$	$\uparrow$	$\uparrow$						cb	^20^
77517	Di/$\upbeta$	$\uparrow$								
CRT1^a^	Di/$\upbeta$	$\uparrow$	$\uparrow$	$\downarrow$	$\downarrow$	$\uparrow$	x	glc, sop^b^	cb, lac	^21^
65191	Di/$\upalpha$	$\uparrow$		$\downarrow$						
67469^a^	Di/$\upalpha$		$\uparrow$			$\uparrow$	x	glc		
69957	Di/$\upalpha$	$\uparrow$	$\uparrow$	$\downarrow$	$\downarrow$				mann, xyl, cb	^38^
69026	Acid	$\uparrow$	$\uparrow$				x	glcUA	galUA	^14^
106330	Acid	$\uparrow$					x	glcUA	galUA	^14^

**Figure 1:**
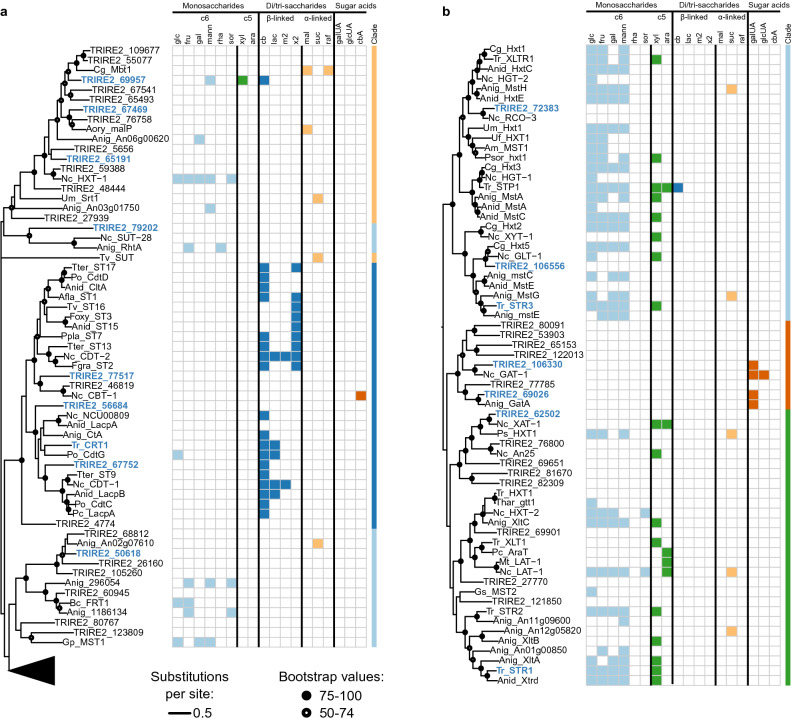
Phylogenetic tree of published sugar transporters from filamentous fungi and putative *T. reesei* sugar transporters. (**a, b**) Phylogenetic tree built from sequence alignment of published fungal sugar transporters and putative *T. reesei* transporters. (**b**) Presents the expansion of the hidden clade from (**a**). Known substrates of the transporters are indicated (References and organisms are listed in Supplementary Table S1). Substrates are divided into monosaccharides (c6: hexoses, c5: pentoses), disaccharides ($\upbeta$- and $\upalpha$-linked) and sugar acids. *T. reesei* transporters which were selected for expression in oocytes are indicated in bold and with color. Scale bar presents substitutions per site and the symbols present bootstrap support values from 100 runs. Abbreviations: glc = d-glucose, fru = d-fructose, gal = d-galactose, mann = d-mannose. rha = l-rhamnose, sor = l-sorbose, xyl = d-xylose, ara = l-arabinose, cb = cellobiose, lac = lactose, m2 = mannobiose, x2 = xylobiose, mal = maltose, suc = sucrose, raf = raffinose, galUA = d-galacturonic acid, glcUA = d-glucuronic acid, cbA = cellobionic acid. The figure was created with ape (version 5.4-1) package for R (version 4.0.3)^79,84^.

Clade refers to clades identified from Fig. 1. Significantly differential expression (DE) by carbon sources or by transcription factors was inferred from published studies^23–25,29,36^. Cel/Lac = DE on cellulose or lactose versus d-glucose^36^, Lac = DE on lactose versus glycerol or d-glucose^23^, XYR1 = DE on cellulose or $\upalpha$-sophorose between $\Delta$*xyr1* and parental QM9414 strains^24^, ACE3 = DE on lactose between $\Delta$*ace3* and parental QM6a strains^25^, CRE1 = DE on d-glucose between $\Delta$*cre1* and parental QM9414 strains^29^. H$^{+}$ indicates symport activity detected in this study. Abbreviations as in Fig. 1.

^a^Sequence differs from that listed in the QM6a genome assembly (See Materials and methods).

^b^$$\upalpha $$ -sophorose.

## Results and discussion

### Selection of *T. reesei* sugar transporters for functional studies

Phylogenetic analysis was used to identify interesting transporters for the functional studies. Putative *T. reesei* sugar transporters were chosen for sequence alignment with previously characterized fungal sugar transporters and a phylogenetic tree was built from the alignment (Fig. 1). We further annotated the tree with substrates known to be transported by the already characterized transporters, which allowed us to assign distinct clades for different substrates. Clades were seen for example for $\upbeta$-linked disaccharide transporters (Fig. 1a, middle part) and sugar acid transporters (Fig. 1b, middle part). A less defined clade containing $\alpha$-linked disaccharide transporters was also visible (Fig. 1a, upper part). Monosaccharide transporters were the most abundant type of transporter in the tree (Fig. 1a lower part, b upper part), which is not surprising since d-glucose is the preferred carbon source for *T. reesei*. Some clades of monosaccharide transporters seemed to be enriched in their ability to transport pentose sugars (Fig. 1b, lower part).

We selected 16 transporters for further analysis (Table 1). These transporters represented different clades of the phylogenetic tree shown in Fig. 1. Although several of these transporters had been published before, we hypothesized that electrophysiological analysis could provide new insights into their function. To complement the phylogenetic analysis, we surveyed published literature about regulation of gene expression by different carbon sources^23,36^, or by the major transcription factors linked to cellulase expression (XYR1, ACE3) or carbon catabolite repression (CRE1)^24,25,29^. Many of the selected transporters had been identified to be significantly upregulated upon growth on inducing carbon sources, cellulose and lactose (Table 1). Interestingly, Trire2_106556 showed the opposite pattern as it was downregulated when the fungus was grown on these carbon sources. Many transporters were downregulated in strains lacking either of the cellulase regulators XYR1 or ACE3^24,37^, with CRT1^19,21,23^ and Trire2_69957^38^ being regulated by both. Several transporters were also upregulated on d-glucose in the absence of carbon catabolite repression regulator CRE1, which suggests that these transporters participate in the transport of sugars other than d-glucose.

The selected transporters were cloned into a *X. laevis* expression vector, and mRNA generated from these constructs was injected into *X. laevis* oocytes. In addition to the transporters listed in Table 1, we included *Neurospora crassa* cellodextrin transporters CDT-1 and CDT-2 as positive and negative controls, respectively. CDT-1 has been shown to function via symport mechanism and CDT-2 as a facilitator^39^, and as previously mentioned, TEVC can only be used for transporters which move net charges, such as symporters. The oocytes were screened for transport activity by perfusing them with different sugars while the electrical current generated across the cell membrane was measured continuously. Each transporter was tested with at least 11 different sugars, including hexose and pentose monosaccharides, as well as $\upbeta$- and $\alpha$-linked disaccharides (see Materials and Methods). Some transporters were also tested with additional sugars or sugar acids on the basis of information from the phylogenetic tree (e.g. Trire2_69026 and _106330 with uronic acids).

Of the 16 *T. reesei* and 2 *N. crassa* transporters tested, 8 proved out to be functional in oocytes (Table 1), as sugar-induced currents were seen in oocytes expressing these transporters (Supplementary Figure S1). No sugar-induced currents were seen in oocytes expressing the other transporters, or in those injected with water (data not shown). The direction of current was inwards, indicating flow of positive ions into the cell, which we hypothesized to be caused by sugar/H^+^ symport activity. Of the 8 identified symporters, transport function had been previously demonstrated for 6 (Table 1), and 2 (CDT-1^39^ and CRT1^21^) were known to be symporters.

We did further tests in yeast with the transporters that did not have symport activity in oocytes and which had not been functionally characterized in the literature. Yeast strains expressing these transporters were tested for d-glucose uptake, as shown in Supplementary Figure S2. With this further screening we were able to identify one additional transporter, Trire2_106556, whose expression enabled yeast to uptake d-glucose and grow on multiple hexose sugars (Supplementary Figure S2). After identifying a set of functional transporters, we did some more specific experiments to characterize them.

### Multiple proteins are responsible for d-glucose transport in *T. reesei*

**Figure 2:**
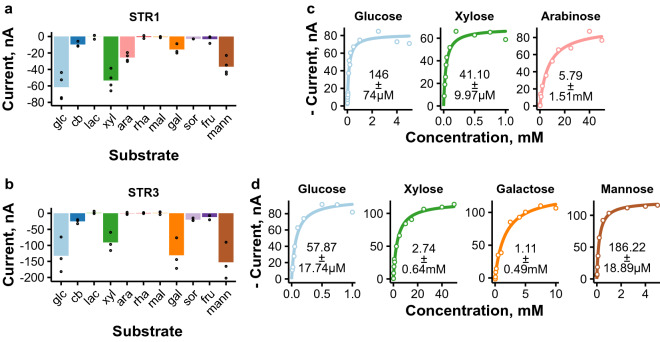
Electrophysiological characterization of d-glucose transporters STR1 and STR3. (**a**, **b**) Selectivity plots of STR1 (**a**) and STR3 (**b**) measured by recording currents in the presence of different sugars in 5 mM concentration (abbreviations as in Fig. 1). Results obtained with − 50 mV test voltage are shown. Points present results obtained from individual oocytes and bars their mean ($n \ge 3$). (**c**, **d**) Sugar transport kinetics of STR1 (**c**) and STR3 (**d**) at − 50 mV. Representative experiments are shown. Lines present the Michaelis Menten kinetics prediction and the *K*_m_ values present the mean and standard deviation from individual oocytes ($n=3$, except $n=5$ for d-glucose kinetics of STR3). The figure was created with ggplot2 package (version 3.3.2) for R^79,85^.

Our analysis identified the previously published d-glucose/d-xylose transporters STR1^7,17^ and STR3^7^ to function via symport mechanism. We further studied their substrate selectivity by recording currents induced by different sugars across multiple test voltages. Representative examples of these I-V curves are shown in Supplementary Figure S3 and the currents obtained at – 50 mV test voltage are shown in Fig. 2a,b. Both transporters proved out to be able to transport multiple monosaccharide sugars, which is in line with previously published results^7^. In addition to previously reported substrates, we identified STR1 to be able to transport l-arabinose and STR3 to be able to transport l-sorbose, which both have been identified as hemicellulase or cellulase inducers in *T. reesei*, respectively^40–42^.

We also analyzed transport kinetics for the main substrates of these transporters (Fig. 2b,c). STR1 had high affinity for both d-glucose and d-xylose, but low affinity for l-arabinose. STR3 had high affinity for d-glucose and d-mannose, but lower affinities for d-galactose and d-xylose. The kinetics for d-glucose and D-xylose have been determined previously in yeast^7^, and our results are mostly in agreement with them (see below). With the oocyte system we could analyze the kinetics at multiple test voltages (Supplementary Figure  S4). With this analysis we discovered that the affinity of both transporters for their substrates decreased as the voltage became more depolarized, except with d-glucose for which clear trends were not seen (Supplementary Figure S5). Transport by STR1 was also found to be more voltage-dependent than that of STR3, as judged by the larger increase in maximum transport rate across the test voltages (Supplementary Figure S6).

**Figure 3:**
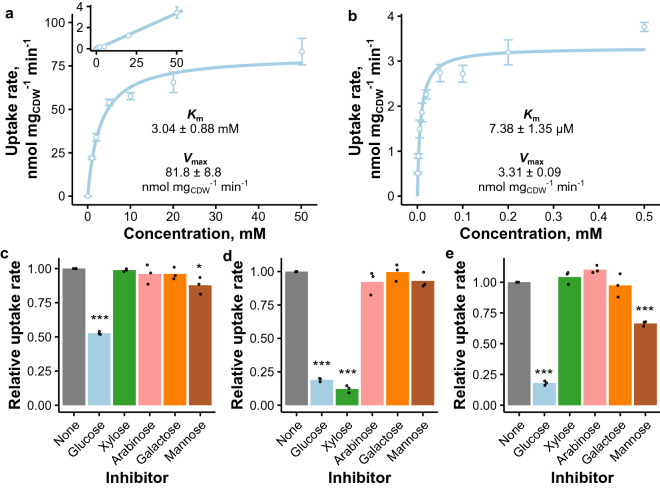
Further d-glucose transporter testing in yeast. (**a**, **b**) Glucose uptake kinetics of GLT1 (**a**) and STP1 (**b**) at pH 6.5. Points and error bars present mean uptake rates and standard deviation between biological replicates ($n=3$, except $n=2$ in the inset in **a**). Lines present the Michaelis-Menten kinetics prediction of the combined values from all biological replicates. The presented *K*_m_ and *V*_max_ values are the mean and standard deviation of kinetics parameters obtained from 3 biological replicates. Inset in **a** shows d-glucose uptake rate of the negative control strain with the line representing linear fit to the data. (**c**–**e**): Inhibition of glucose transport of GLT1 (**c**), STR1 (**d**) or STR3 (**e**) in a competition assay with inhibitors in 5-fold excess (1 vs 5 mM) at pH 6.5. Points present values obtained for biological replicates and bars their mean ($n=3$). Significance in relation to condition without inhibitor was estimated with ANOVA and Tukey’s honest significant difference test (*$:p<$ 0.05, ***$:p<$ 0.005). The figure was created with ggplot2 package for R^79,85^.

One of the transporters identified from the phylogenetic analysis and expression data survey was Trire2_106556, which was here named GLT1 (**gl**ucose **t**ransporter **1**). It clustered together with low-affinity d-glucose transporters from *N. crassa* and *C. graminicola*^8,11^ (Fig. 1), and, in contrast to the majority of the selected transporters, it was upregulated on d-glucose (Table 1 and Ref.^43^). Although both yeast (Supplementary Figure S2) and oocytes (data not shown) expressing this transporter were able to uptake d-glucose, no sugar-induced currents were detected with TEVC from oocytes expressing this transporter. Therefore we hypothesized that it functions as a passive facilitator. Analysis of the transport kinetics in yeast proved that GLT1 is a low-affinity d-glucose transporter (Fig. 3a). The results contradict a recent study where no transport activity was detected in yeast strain expressing GLT1^18^. However, since the used amino acid sequences were identical, we can’t offer a reason for this discrepancy. In the same conditions, a linear relationship between the uptake rate and d-glucose concentration was observed in the negative control strain (Fig. 3a), which is consistent with earlier reports^12^. The used strain was based on the EBY.VW5000 background, in which some low-affinity d-glucose uptake is observed due to the *SNF3* deletion^44^.

The function of GLT1 is in line with its homology to low-affinity d-glucose transporter GLT-1 from *N. crassa* (Fig. 1). The d-glucose transport system of *N. crassa* also includes two high-affinity transporters (HGT-1 and -2)^8^. Their *T. reesei* homologs are facilitators STP1^19,45,46^ and XLTR1^18^ (Fig. 1), of which the latter appears to be expressed only at low levels based on published transcriptome data sets^22,23^. On the other hand, STP1 has been found to be expressed constitutively across multiple carbon sources (Table S1 of Ref.^36^). We expressed STP1 in yeast to study its d-glucose transport kinetics, since as a passive facilitator^46^ it could not be studied with TEVC. As hypothesized from the phylogenetic analysis, STP1 proved out to be high-affinity d-glucose transporter (Fig. 3b).

We also did competition experiments for GLT1, STR1 and STR3 with the yeast system to further investigate their substrate specificity. d-glucose transport by GLT1 was not inhibited significantly by any of the tested sugars in 5-fold excess except by d-glucose itself (as expected) and slightly by d-mannose (Fig. 3c), suggesting that d-glucose is the primary substrate. Regarding STR1, its d-glucose transport activity was heavily inhibited by d-xylose (Fig. 3d), which is in line with its high affinity for this substrate (Fig. 2c). d-glucose transport by STR3 was inhibited by D-glucose, d-mannose and d-galactose (Fig. 3e), as expected from the kinetics results (Fig. 2d). We did not include STP1 in the competition experiments, since a similar experiment had been done previously^46^. In the previous study, the transport of a fluorescent d-glucose analog was found to be inhibited severely by d-glucose, d-mannose and d-fructose, less severely by d-xylose and only slightly by l-arabinose when the competing sugars were in 10-fold excess^46^.

The discovery of low-affinity d-glucose transporter GLT1, identification of the symport mechanism and new substrates for STR1 and STR3 and the kinetic characterization of two important d-glucose transporters (STP1, GLT1) provide new information about the d-glucose transport system of *T. reesei*. Although our kinetics analysis for STR1 and STR3 was mostly in agreement with a previous study^7^, as shown in Supplementary Table S2, we noticed that only low affinity d-xylose transport activity had previously been described for STR1. However, we observed that the d-xylose kinetics from a previous report appeared to exhibit biphasic behavior, with high-affinity (0–5 mM) and low affinity (5–50 mM) components (Additional File 7 in Ref.^7^). Since the previously reported kinetics parameters were from a curve fitted to the whole concentration range, a separate fit for the high-affinity component would have resulted in a significantly lower *K*_m_ value. The high affinity for d-xylose was further supported by the competition experiment, which showed that d-glucose transport by STR1 was heavily inhibited by d-xylose. The XYR1-dependent upregulation of STR1 in cellulase-inducing conditions (Table 1) as well as upon growth on d-xylose, and the severe growth defect of the *str1* deletion strain on d-xylose and l-arabinose, but not on d-glucose, further support its physiological role as a pentose transporter rather than as a d-glucose transporter^7,17^. Indeed, the l-arabinose transport activity discovered here could explain the growth defect of $\Delta$*str1* strain on this sugar^17^.

### Cellodextrin transporters CDT-1 and CRT1 have differences in substrate selectivity and in their affinity for protons

**Figure 4:**
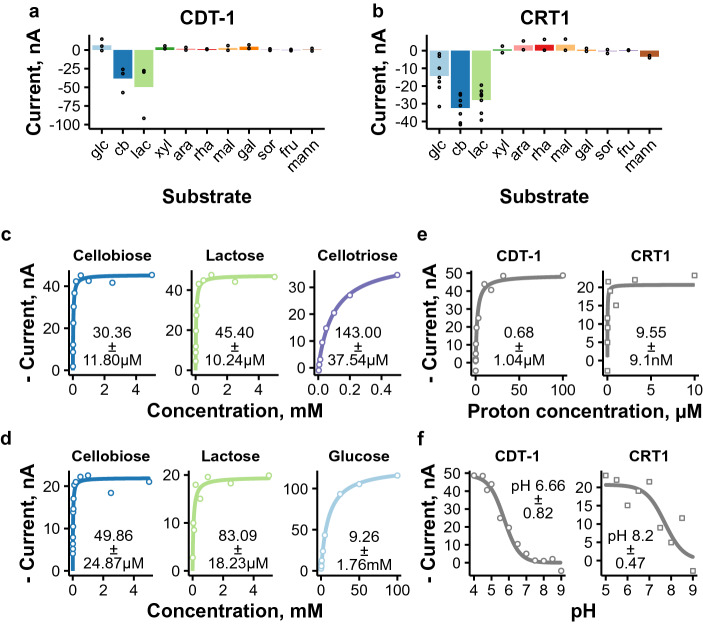
Electrophysiological characterization of cellodextrin transporters CDT-1 and CRT1. (**a**, **b**) Selectivity plots of CDT-1 (**a**) and CRT1 (**b**) measured by recording currents in the presence of different sugars in 5 mM concentration (abbreviations as in Fig. 1). Results obtained with − 50 mV test voltage are shown. Points present results obtained from individual oocytes and bars their mean ($n \ge 2$). (**c**, **d**) Sugar transport kinetics of CDT-1 (**c**) and CRT1 (**d**) at − 50 mV. (**e**, **f**) Dependence of cellobiose transport by CDT-1 and CRT1 on proton concentration, with the proton concentration presented on linear (**e**) and log scale (**f**). Lines in panels (**c**–**f**) present the Michaelis Menten kinetics prediction, and the *K*_m_ values present mean and standard deviation from individual oocytes ($n=3$, except $n=4$ for cellobiose kinetics of CRT1). Representative experiments are shown in panels (**c–f**). The figure was created with ggplot2 package for R^79,85^.

Our initial screening identified two previously published cellobiose symporters, CDT-1 and CRT1, to be functional in oocytes. Both are important for cellulase induction. In *T. reesei*, deletion of CRT1 is enough to abolish cellulase induction, whereas in *N. crassa* deletion of both CDT-1 and another cellobiose transporter CDT-2 is required^19,23,47^. Both CDT-1 and CRT1 have been shown to be able to transport cellobiose and lactose when expressed in yeast^21,48^, and this was also seen in oocytes (Fig. 4a,b). Kinetics measurements for these sugars, shown in Fig. 4c,d, revealed results following a similar trend as seen in previous studies, where the kinetics were determined in yeast (Supplementary Table S3). The results showed that cellobiose was transported with higher affinity compared to lactose and that CDT-1 had higher affinity for these two substrates compared to CRT1.

Regarding other substrates, CRT1 could also transport d-glucose, but its affinity for this sugar was about 100–200-fold lower than for cellobiose or lactose. d-Glucose transport has also been observed for its closest homolog in Fig. 1, *P. oxalicum* CdtG^49^. Additional experiments also indicated that CRT1 was able to transport the potent cellulase inducer $\alpha$-sophorose (Supplementary Figure  S7). This observation could provide explanation for previous reports that have shown that CRT1 is indispensable for $\alpha$-sophorose mediated cellulase induction, although opposite findings have also been reported^19,21,23^. In one published study, CRT1 deletion did not appear to affect $\alpha$-sophorose uptake^19^, which indicates that there might be other transporters for $\alpha$-sophorose and thus makes the understanding of components needed for induction more intriguing. $\alpha$-sophorose transport was not tested with CDT-1 since $\alpha$-sophorose does not function as an inducer in *N. crassa*^47^.

Besides cellobiose and lactose, CDT-1 has been shown to be able to transport cellodextrins cellotriose and cellotetraose^48^. We were interested in determining kinetics for these longer sugars, as this was not addressed in previous studies. Cellodextrins are probably rather abundant in the natural breakdown products of cellulose. Thus it is important to understand how short the enzyme-degraded sugars need to be in order to be taken up by the fungal cell, since the cellulose filaments are thousands of d-glucose units long. As expected based on previous reports, cellotriose was transported by oocytes expressing CDT-1 (Supplementary Figure S7). The affinity of CDT-1 for cellotriose was high, although lower than for cellobiose and lactose (Fig. 4c). We did not detect cellotetraose transport in oocytes, and thus we were unable to do the kinetic analysis for this sugar. Previously reported results from growth and inhibition tests have indicated that cellotetraose is transported, although less efficiently than cellotriose^21,48^. Therefore, the cellotetraose concentration in our assay could have been too low to detect transport.

We also analyzed the pH-dependence of these transporters by measuring the currents induced by 5 mM cellobiose as a function of proton concentration (Fig. 4e). CRT1 had higher affinity for protons than CDT-1, and thus it is able to function in a broader pH range than CDT-1 (Fig. 4f). We are aware of a single fungal transporter whose pH-dependence has been characterized before with TEVC (*U. maydis* Srt1)^33^, and its *K*_m_ for protons is quite similar to that obtained for CRT1 (CRT1: pH 8.2 ± 0.47 vs Srt1: pH 7.7 ± 0.31). The observed differences in the affinity for protons are interesting, since both *N. crassa* and *T. reesei* are usually grown in slightly acidic conditions and both employ saprotrophic lifestyle. Regarding voltage kinetics, a slight increase in affinity was seen for CRT1 as the voltage became more depolarized (Supplementary Figure S8).

### Identification of novel low-affinity hexose transporters Trire2_50618 (FRT1) and Trire2_67469 (MLT1)

**Figure 5:**
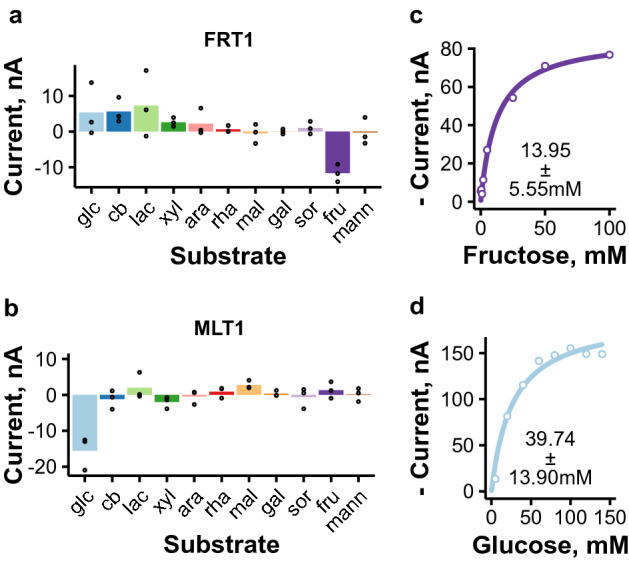
Electrophysiological characterization of hexose transporters FRT1 and MLT1. (**a**, **b**) Selectivity plots of FRT1 (**a**) and MLT1 (**b**) measured by recording currents in the presence of different sugars in 5 mM concentration (abbreviations as in Fig. 1). Results obtained with − 50 mV test voltage are shown. Points present results obtained from individual oocytes and bars their mean ($n=3$). (**c**, **d**) d-fructose transport kinetics of FRT1 (**c**) and d-glucose transport kinetics of MLT1 (**d**) at − 50 mV. Representative experiments are shown. Lines present Michaelis Menten kinetics prediction and the *K*_m_ values present mean and standard deviation from individual oocytes ($n=6$ for Trire2_50618, $n=4$ for Trire2_67469). The figure was created with ggplot2 package for R^79,85^.

Two novel monosaccharide transporters, Trire2_50618 and _67469, were identified from the initial screening (Supplementary Figure S1) as being able to transport d-fructose and d-glucose, respectively. They were named FRT1 and MLT1, for **fr**uctose **t**ransporter 1 and **m**a**l**tose **t**ransporter-like protein **1**, respectively. More detailed selectivity studies indicated that these two transporters appeared to be specific for their single substrates (Fig. 5a,b). The currents elicited by FRT1 and MLT1 were lower than those elicited by the other transporters at the 5 mM concentration used in the initial screening. This observation was explained by kinetics measurements, from which we discovered that these transporters have low affinities for their substrates (Fig. 5c,d). Regarding voltage-dependence of kinetic parameters, the *K*_m_ values as a function of voltage showed opposite trends for FRT1 and MLT1 (Supplementary Figure  S5), and the maximum transport rate by FRT1 was more dependent on voltage than that of MLT1 (Supplementary Figure S6).

The discovery of these new sugar transporters provides further information about the sugar transport system of *T. reesei*. Interestingly, the affinities observed for these transporters are lower than what have typically been observed for fungal hexose transporters (Supplementary Table S2), although low/moderate-affinity yeast hexose transporters have *K*_m_ values in this range^5^. The observed low affinities might indicate that d-fructose and d-glucose are not the main substrates of these transporters. Regarding MLT1, this hypothesis is supported by its homology to $\alpha$-linked disaccharide transporters (Fig. 1) and by its upregulation in the absence of CRE1 (Table 1). Interestingly, two putative maltose transporters (Trire2_65191 and _59388) have been either lost or mutated in *T. reesei* hyperproducer strain RUT-C30, which grows poorly on maltose^50,51^. The same strain has also a mutation in regulator BglR, which is involved in both d-glucose and maltose utilization^52^. However, the improved growth of RUT-C30 on d-glucose is not consistent with BglR-negative phenotype^50,52^, which could indicate that BglR is still functional in this strain, and that the poor growth on maltose is indeed caused by defects in maltose transport.

FRT1, on the other hand, had some homology to characterized d-fructose transporters (Fig. 1), which themselves are homologs of the *Saccharomyces pastorianus* d-fructose/l-sorbose symporter Fsy1^53–55^. However, as shown in Supplementary table S2, its affinity for d-fructose is lower than what has been observed for these transporters (*Botrytis cinerea* FRT1, *Aspergillus niger* 296054 and 1186134^53,54^). In Fig. 1, FRT1 is grouped under different clade than these transporters, as was also previously shown for An02g07610^56^, which is in the same clade as FRT1 in Fig. 1. An02g07610 has been found to be upregulated in response to pectin-related carbon sources (d-galacturonic acid, l-rhamnose, d-galactose, l-arabinose), although one report indicated that it functions as a sucrose transporter in yeast^56,57^. The observed specificity of FRT1 for d-fructose, an easily metabolizable monosaccharide, is interesting since FRT1 has consistently been found to be upregulated in cellulase-inducing conditions^22,36,58^ and to be downregulated in the presence of d-glucose in CRE1-dependent manner^29,43^. Additionally, FRT1 has also been found to be differentially phosphorylated in response to cellulase-induction^59^.

### Uronic acid transporters Trire2_106330 (GAT1) and Trire2_69026 (GAT2) prefer different substrates

Our initial screening identified two transporters capable of transporting d-galacturonic and d-glucuronic acids. d-Galacturonic acid is the primary component of the plant cell wall polysaccharide pectin. Pectin-rich sidestreams from e.g. fruit processing industry have gained interest as feedstock for bioprocesses, since they are available in large quantities and contain lower amounts of lignin than lignocellulose biomass^14^. d-Glucuronic acid, on the other hand, is present in the hemicellulose fraction of lignocellulose biomass. Both of these transporters had been identified in a previous study, but apart from identifying their transport activity in yeast, no characterization had been done on them^14^. Since our initial screening identified them to be functional in oocytes, we set out to investigate them more thoroughly.

**Figure 6:**
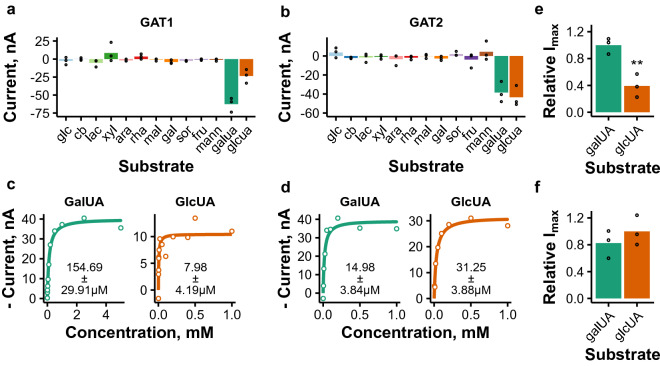
Electrophysiological characterization of uronic acid transporters GAT1 and GAT2. (**a**, **b**) Selectivity plots of GAT1 (**a**) and GAT2 (**b**) measured by recording currents in the presence of different sugars and sugar acids in 5 mM concentration (abbreviations as in Fig. 1). Results obtained with − 50 mV test voltage are shown. Points present results obtained from individual oocytes and bars their mean ($n=3$). (**c**,**d**) Sugar acid transport kinetics of GAT1 (**c**) and GAT2 (**d**) at − 50 mV. Representative experiments are shown. Lines present the Michaelis Menten kinetics prediction and the *K*_m_ values present the mean and standard deviation from individual oocytes ($n=3$). (**e**, **f**) Relative *I*_max_ values for GAT1 (**e**) and GAT2 (**f**) at − 50 mV. *I*_max_ values from individual oocytes were normalized to the mean *I*_max_ value of the substrate with the highest *I*_max_ value for each transporter. Points present results from individual oocytes and bars their mean ($n=3$). Significance was estimated with Student’s *t*-test (**$:p<0.01$). The figure was created with ggplot2 package for R^79,85^.

The two uronic acid transporters seemed to be specific for their substrates (Fig. 6a,b), with d-galacturonic acid inducing higher currents in Trire2_106330 (here named GAT1, for **ga**lacturonic acid **t**ransporter **1**) than d-glucuronic acid. Kinetics experiments indicated that GAT1 has higher affinity for d-glucuronic acid than for D-galacturonic acid (Fig. 6c). Conversely Trire2_69026 (here named GAT2) has higher affinity for d-galacturonic acid, but the difference is not as large as with GAT1 (Fig. 6d). GAT1 had significantly higher *I*_max_ value for D-galacturonic than for d-glucuronic acid (Fig. 6e), in contrast to GAT2 for which the *I*_max_ values were not significantly different (Fig. 6f). Of the transporters studied here, GAT1 was the only one which had significantly different *I*_max_ values for different substrates across all test voltages. This difference might allow the transport activity to be tuned to different sugar acids released from different plant cell wall polysaccharides. Regarding voltage-dependence of the kinetics, GAT2 was discovered to be more voltage-dependent than GAT1 (Supplementary Figure S6), and its affinity was found to decrease as the voltage became more depolarized (Supplementary Figure S5).

To our knowledge, only two fungal uronic acid transporters have been kinetically characterized in the literature, although kinetic parameters are only available for d-galacturonic acid^14,60^. The affinities of GAT1 and GAT2 for d-galacturonic acid lie between the previously characterized transporters from *N. crassa* and *A. niger* (Supplementary Table S3). For the first time, two uronic acid transporters have been characterized from the same fungal species and interestingly they had different preferences for d-galacturonic and d-glucuronic acids. More studies would be needed to investigate if they are regulated differently, since these sugar acids are found from different plant cell wall polysaccharides. Although pectinolytic regulators (such as GaaX, GaaR and RhaR of *Aspergilli*^61^) have not yet been characterized from *T. reesei*, l-arabinose/d-galactose regulator ARA1 has been shown to regulate genes related to both hemicellulase and pectin metabolism, as l-arabinose is present in both polymers^41^.

## Conclusions

The purpose of our study was to gain better understanding of the sugar transporter repertoire of *T. reesei*. Phylogenetic analysis was used to identify a reasonably sized set of transporters for the functional analysis, which provided a substantial amount of new information about *T. reesei* sugar transporters. All in all, transport activity was described for the first time for three transporters (GLT1, FRT1, MLT1), and we were also able to identify many new functionalities for transporters which had been identified previously.

The studied set of transporters was diverse, and we could identify transporters for all the different classes of sugars mentioned in Fig. 1, except for $\alpha$-linked disaccharides. It is possible that some of the transporters for which we didn’t detect transport activity might still exhibit it. There could have been problems with protein expression, folding or insertion to the membrane, but we did not address or try to troubleshoot these possibilities. Some membrane transporters require ancillary proteins to function or to be correctly localized^62,63^, but to our knowledge this has not been observed in fungal sugar transporters. There are also many possible substrates that we didn’t analyze (e.g. polyols). Additionally, some transporters appear to exhibit their activity only on a certain concentration range, which we might have missed^64^.

Besides providing information about the physiology of the fungus, these results could have practical applications. For example, deletion of d-glucose transporter(s) has resulted in increased protein production in both *N. crassa* and *T. reesei*^8,19^. Double deletion of high-affinity d-glucose transporters STP1 and STR3 could be attempted for this purpose, as has been done in *N. crassa*^8^. Since *T. reesei* has been modified to produce high amounts of protein with d-glucose as the carbon source^65–67^, overexpression of GLT1 could be also attempted to increase the protein production in these strains. Nevertheless, protein production is conventionally done on lactose-based media, and thus manipulation of $\upbeta$-linked disaccharide transport could be also attempted. As a practical example, overexpression of CRT1 has been indeed shown to result in earlier onset of cellulase production or in higher cellulase expression^19,21,23^.

Regarding transport kinetics, the majority of the identified transporters exhibited high or moderate affinity for their primary substrates. Exceptions were MLT1 and FRT1, which had considerably low affinity when compared to other fungal transporters capable of transporting these sugars (Supplementary Table S2). As in *N. crassa*^8^, the d-glucose transport system of *T. reesei* appears to include high-affinity (STP1, STR3) and low/moderate-affinity (GLT1) transporters. In addition to these transporters, three additional d-glucose transporters have been identified from *T. reesei* (STR1^7,17^, STR2^7^, XLTR1^18^), but their expression profile does not support their involvement in the bulk of d-glucose transport.

This study, along with two previously published studies^33,34^, employed *X. laevis* oocytes for the electrophysiological characterization of fungal sugar transporters. Although there were differences in the *K*_m_ values determined in yeast and oocytes, the general trend (low or high affinity) was similar between both as shown in Supplementary Figure S9. These differences could be caused by differences in membrane potential or in the lipid composition of the cell membrane. Since many of the characterized fungal sugar transporters utilize the proton symport mechanism (Supplementary Tables S2 and S3), they could be studied further with this system. Additionally, since the genomes of saprophytic fungi contain many uncharacterized sugar transporters, electrophysiological methods could be used for the identification of transport activities from the thus far unknown transporters. Since robotic systems for oocyte injection and TEVC analysis are available^68^, high-throughput screening would be also possible.

## Materials and methods

### Microbial media, cultivation and chemicals

Yeast strains were cultured in YP (1% yeast extract, 2% peptone) or synthetic complete (0.67% yeast nitrogen base without amino acids, SC-Ura drop-out mix) medium with 2% glycerol and 2% ethanol as carbon sources unless otherwise mentioned^69^. Both media were supplemented with 2% agar for preparing solid media. Yeast was grown in $30\,^{\circ }$C with 230 rpm shaking in 20 mL volume in 100 mL flasks or in 50 mL volume in 250 mL flasks. Growth curves were recorded with Bioscreen C incubator (Oy Growth Curves Ab, Helsinki, Finland) as described before^21^.

Chemicals were obtained from Merck KGaA (Darmstadt, Germany) and molecular biology reagents from Thermo Fisher Scientific (Waltham, MA, USA) unless otherwise mentioned. Lactose was obtained from VWR (Helsinki, Finland), and l-rhamnose and l-sorbose were obtained from Fluka (Charlotte, NC, USA). Cellotriose and cellotetraose were obtained from Carbosynth (Compton, Berkshire, UK) and $\alpha$-sophorose from SERVA Electrophoresis GmbH (Heidelberg, Germany).

### Molecular biology methods

High-fidelity PCR was done with KAPA HiFi polymerase (Roche, Basel, Switzerland) and colony PCR with DreamTaq polymerase. Restriction digestions were performed with restriction enzymes from Thermo Fisher Scientific or NEB (Ipswich, MA, USA). *E. coli* transformations were done with electroporation and yeast transformations with the LiAc/ssDNA/PEG method described by Gietz and Woods^70^. *In vitro* transcription was done with mMessage mMachine T7 kit. Primers were obtained from Thermo Fisher Scientific and they are listed in Supplementary Table S4. *Escherichia coli* strain Top10 was used for cloning and for storing the plasmids.

### Expression of transporters in yeast

For the yeast expression, synthetic genes were obtained with codon-optimization for yeast (GeneArt, Thermo-Scientific), with the exception of Trire2_69026 which was obtained from *T. reesei* cDNA. The amino acid sequences for gene synthesis were obtained from the *T. reesei* QM6a genome annotation (version 2.0)^71^, except for Trire2_62380 (STR3), _3405 (CRT1), _67752 and _67469. For Trire2_62380, _3405 and _67752, we used the genes from the RUT-C30 genome assembly^72^ (TrireRUT-C30_95062, _109243, and _79984, respectively). RUT-C30 versions of Trire2_3405 and _67752 have been shown to be functional in previous studies^20,21^. Sequence for Trire2_67469 was manually annotated based on a homolog identified from other species of *Trichoderma*, as shown in Supplementary Figure S10a. Similarly, we identified an alternative version for Trire2_56684, which was expressed in addition to the QM6a version (Supplementary Figure S10b). For Trire2_72383, we expressed both RUT-C30 and QM6a version. All constructs are listed in Supplementary Table S5.

The synthetic genes were cloned to yeast expression vector (*URA3*, *CEN6*/*ARS4*, *PGK1* promoter, *ENO1* terminator) with the yeast MoClo toolkit (parts MoClo parts pYTK-2, -11, -51, -67, -74, -81, -84)^73^. The resulting constructs were sequenced with primers SaSS-19 and -20. The gene coding for Trire2_69026 was amplified from *T. reesei* QM6a cDNA with primers SaSS-75–76, and ligated to B2159 vector (derived from pYX212 as described in Ref.^74^) which had been digested with *Eco*RI and *Bam*HI. The resulting construct was sequenced with primers TPI1_P_long and TPI1_T_long.

The expression plasmids were transformed into yeast strain ySS1, which as an EBY.VW5000 derivative lacks the endogenous hexose transporters and additionally expresses *N. crassa* intracellular $\upbeta$ -glucosidase GH1-1^21^. ySS1 was pre-grown on YPGE and the transformation reactions were plated on SCGE-Ura medium.

### Construction of plasmids for *X. laevis* expression

To express the synthetic genes in *X. laevis* oocytes, the oocyte expression vector Pol1^75^ was modified to be compatible with the yeast MoClo system (Havukainen *et al.*, submitted). The MoClo-compatible version of Pol1 was digested with *Bsa*I and the two resulting fragments (Pol1 contains one endogenous *Bsa*I site) were gel purified. The backbone fragments were ligated with T4 ligase (NEB) with the genes which were liberated from the original GeneArt pMA-T plasmids with *Bsa*I. The constructs were sequenced with primers SaSS-57 primer and T7 promoter primer.

The genes coding for Trire2_69026, _72383, _77517 and _79202 were obtained from *T. reesei* cDNA for *X. laevis* expression. Trire2_69026 was amplified from plasmid pSS87 (Supplementary Table S5) with primers SaSS-95–96 and the amplicon was digested with *Bam*HI and *Eco*RI and ligated to Pol1 vector digested with the same enzymes. The genes coding for Trire2_72383, _77517 and _79202 were amplified from cDNA prepared from *T. reesei* QM6a with primers PP-203–204 and PP-209–212. Trire2_72383 amplicon was digested with *Bam*HI and *Hind*III and ligated to *X. laevis* expression vector pMJB08 (pol1 derivative with various tags^76^) which had been digested with the same enzymes. Trire2_77517 and _79202 expression vectors were constructed similarly, except that for Trire2_77517 the amplicon and vector were cut with *Bam*HI and *Eco*RI, and for Trire2_79202 they were cut with *Xba*I and *Hind*III. We noted that the Trire2_79202 sequence obtained from cDNA differed from the QM6a sequence in that the second intron was missing (Supplementary Figure S10c).

### Preparation of mRNA and *X. laevis* oocyte injection

Oocytes were obtained via surgery as described by Clemencon *et al.*^77^. Briefly, adult female *X. laevis* frogs were anaesthetized by immersion in ice/water slurry containing 1 g/L 3-aminobenzoate methanesulfonate and the oocytes were removed with surgery. After removing the oocytes, the cuts were sutured, and after recovering from anesthesia the frog was placed in isolation tank to recover for 1 week. During the studies, each of the used frogs was subjected to surgery only once. All experiments using the *X. laevis* animals were in accordance with the Swiss Animal Welfare law, reported according to applicable ARRIVE guidelines^78^ and approved by the local Veterinary Authority (Amt für Veterinärwesen Kantons Bern; Permit Number: BE60/2018).

After the surgery, the oocytes were suspended in modified Barth’s medium with Ca^2+^ (MBM+Ca^2+^; 88 mM NaCl, 1 mM KCl, 2.4 mM NaHCO_3_, 1.57 mM MgSO_4_$\cdot$7 H_2_O, 0.66 mM NaNO_3_, 0.75 mM CaCl_2_, 10 mM HEPES) which was supplemented with penicillin and streptomycin. The oocyte sacs were separated to 3-4 mm pieces with forceps. Oocytes were defolliculated by washing with MBM-Ca^2+^ (MBM+Ca^2+^ without CaCl_2_), after which they were suspended to MBM-Ca^2+^ with 4 g/L collagenase (NB4, SERVA Electrophoresis GmbH, Heidelberg, Germany) and incubated in rocking shaker for 1 hour. After the incubation, the washing and collagenase incubation steps were repeated. Finally the oocytes were washed with MBM+Ca^2+^ and healthy stage V-VI oocytes were picked.

The pol1-based plasmids containing the transporter CDSs were linearized with *Nhe*I and *in vitro* transcribed into mRNA. After the transcription the product was purified with LiCl precipitation according to the kit instructions. The oocytes were injected with 10–30 ng mRNA in 50 nL volume or with the same volume of water. The injection was done with Nanoject II microinjector (Drummond Scientific, Broomall, PA, USA). After injection the oocytes were suspended in MBM+Ca^2+^ and incubated in $17\,^{\circ }C$ for 3–7 days.

### Electrophysiology experiments with *X. laevis* oocytes

Two-electrode voltage clamp (TEVC) technique was used for the electrophysiological characterization. In this method, the membrane potential of the oocyte is clamped to a specific voltage and the transport is measured by recording the substrate-induced changes in the membrane current^32^. The measurements were done on a TEVC setup consisting of 2-channel perfusion system, OC-725C Oocyte clamp amplifier (Warner Instruments, Hamden, CT, USA) and Axon Digidata 1440A digitizer (Molecular Devices, San Jose, CA, USA). The system was calibrated with oocyte model cell. Microelectrodes filled with 3 M KCl and with resistance between 0.5–5 M$\Omega$ were used. pClamp software suite (version 10.2, Molecular Devices, https://www.moleculardevices.com/) was used for the analysis. ND-96 buffer without sodium (100 mM choline, 2 mM KCl, 1 mM CaCl_2_$\cdot$2H_2_O, 1 mM MgCl_2_$\cdot$6H_2_O, 3 mM HEPES) was used for the experiments. Each experiment was done with at least three oocytes derived from at least two different frogs, except for some sugars in CRT1 and CDT-1 selectivity experiments where only 2 oocytes were used. Each transporter was tested at least with 11 different sugars, including monosaccharides and disaccharides (glc, fru, gal, mann, rha, sor, xyl, ara, cb, lac, mal; abbreviations as in Fig. 1).

For the current trace recordings, the oocytes were perfused with ND-96 (pH 7.4) and clamped at − 50 mV. For each sugar, the oocyte was first perfused with ND-96 (pH 7.4) until the current stabilized, then with ND-96 (pH 5.5) until the current stabilized and finally with ND-96 (pH 5.5) containing 5 mM sugar. Afterwards the current was recovered to baseline by perfusing with ND-96 (pH 7.4). The resulting current traces were recorded with Axoscope program of the pClamp software suite.

The I–V curves were recorded with Clampex program of the pClamp software suite. The oocytes were clamped at − 50 mV, perfused with the test solution until the current stabilized and then 200 ms step-wise changes of membrane potential from − 150 mV to 50 mV were applied. For each measurement, a recording was done first with buffer alone and then with the same buffer which contained the sugar to be analyzed. The steady-state currents obtained in the absence of sugar were subtracted from the currents obtained in the presence of sugar for each of the tested voltages. Between measurements the oocyte was stabilized by perfusing with pH 7.4 buffer.

The selectivity was measured by recording I–V curves with 5 mM substrate solutions prepared in ND-96 pH 5.5. Sugar acid solutions were adjusted to pH 5.5 with KOH. For the kinetics experiments I–V curve recordings were done with different sugar concentrations with pH 5.5 buffer. For the pH-dependence experiments of CDT-1 and CRT1, I–V curve recordings were done with ND-96 (with 5 mM cellobiose) adjusted to different pH values. The kinetics parameters for sugars and protons were calculated by fitting the currents as a function of substrate concentration to Michaelis-Menten equation (Eq. 1), where $I_{max}$ = maximum current, [*S*] = substrate concentration and $K_m$ = Michaelis constant. The fitting was done with the nls function with the default Gauss-Newton algorithm. The nls function belongs to package stats which is part of R^79^. The *K*_m_ values are presented as mean ± standard deviation from at least three different oocytes. For the analysis of voltage-dependence of kinetics shown in Supplementary Figures S4, S6 and S8, we normalized the currents to the highest negative current obtained at − 50 mV during the experiment.

1 $$\begin{aligned} I = \frac{I_{max} [S]}{[S] + K_m} \end{aligned}$$

### Uptake experiments

Uptake experiments were done as previously^21^. Briefly, ySS1-based yeast strains were inoculated into 20 mL SCGE-Ura medium and grown for 2 days. Then the strains were inoculated into 50 mL of the same medium and grown into exponential phase. Cells were harvested by centrifugation, washed with water and resuspended to uptake buffer (100 mM K-PO_4_, pH 6.5). Rabiolabeled d-glucose (MC 144, Lot 945-006-256-A-20180329-DNG, Moravek Biochemicals, Brea, CA, USA) was diluted with ordinary sugar to reach the desired concentration and radiospecific activity. Aliquots of the yeast suspensions and the label were incubated 5–10 min at 28 $^{\circ}$C before starting the reaction. Reaction mixture consisted of 40 $\upmu$L cell suspension and 20 $\upmu$L of the label in a cone-shaped glass tube. Reaction was stopped after desired time by adding 10 mL ice-cold water to the reaction and filtering the resulting mixture. The tube was washed with same amount of water, which was also filtered. The filter containing the yeast was suspended to 4 mL of Ultima Gold XR liquid scintillation cocktail (PerkinElmer, Waltham, MA, USA). The samples were counted with TriCarb 2810 TR scintillation counter (PerkinElmer). Blank samples were measured similarly, except that 10 mL water was added to yeast suspension before the label, and after the addition of the label the mixture was filtered immediately. The chosen reaction times were ensured to be on the part of the curve which was linear in respect to time.

Uptake rate was determined by substracting the counts (as counts per minute, cpm) obtained for the blank samples from the actual samples, and by dividing the blank-corrected counts with the radiospecific activity of the label (cpm/nmol), the amount of yeast used (OD) and by the reaction time (min). OD values were further converted to CDW values using a previously published correlation factor for ySS1^21^. d-glucose uptake kinetics were fitted to equation 2 individually for each biological replicate (*n*=3), and the values are presented as mean ± standard deviation from these fits. The negative control strain expressed *T. reesei* intracellular $\upbeta$-glucosidase CEL1a from a similar expression plasmid^21^.

2 $$\begin{aligned} V = \frac{V_{max} [S]}{[S] + K_m} \end{aligned}$$

### Phylogenetic analysis

Published fungal sugar transporters and putative *T. reesei* sugar transporters were selected for the phylogenetic analysis. A List of putative *T. reesei* sugar transporters was obtained from Ref.^1^. From this list we excluded some transporters based on their transporter classification database (TCDB) family^80^, if the description indicated that the predicted substrate was not sugar, polyol or quinate (Trire2_106118, _121441, _44175, _45852, _77552, _81389, _45868, _60988, _82037). Some were further exluded based on transmembrane prediction, which indicated that they didn’t possess the characteristic 12 transmembrane domain topology of major facilitator superfamily transporters (Trire2_5890, _75021, _80058, _61278, _60086). Additionally, we included putative transporter Trire2_79202 since it has been hypothesized to be a lactose transporter^28^.

The amino acid sequences of the transporters were obtained from GenBank or Uniprot. Their accession numbers with references for the transported substrates are listed in in Supplementary Table S1. We did not consider deletion phenotypes alone as definite evidence of transport function (as with e.g. Tr_HXT1^6^, Nc_SUT-28^27^), and thus these substrates were not included in the figure. Sequence alignment was done with MUSCLE^81^, and the phylogenetic analysis was performed with maximum likelihood method with 100 bootstrap runs with partial gap removal (95% site coverage cutoff)^82^. Both steps were performed with MEGA X software (version 10.1.1)^83^, and the tree was visualized with ape package for R^84^.

## Supplementary Information

Supplementary Information 1

## Data Availability

The datasets generated during the current study are available from the corresponding author on reasonable request.
