# Supplementary material for: Electrophysiological characterization of a diverse group of sugar transporters from Trichoderma reesei
Source: Sci Rep. 2021 Jul 19;11:14678. doi: 10.1038/s41598-021-93552-7 (PMC8290022; doi:10.1038/s41598-021-93552-7)
Supplement: Supplementary file 1 — Supplementary Information 1 [file 41598_2021_93552_MOESM1_ESM.pdf]

Electrophysiological characterization of a diverse group of sugar transporters from *Trichoderma reesei*

Sami Havukainen[1], Jonai Pujol-Giménez[2], Mari Valkonen[1], Ann Westerholm-Parvinen[1], Matthias A. Hediger[2], and Christopher P. Landowski[1, *]

[1]Protein Production Team, VTT Technical Research Center of Finland Ltd, Tietotie 2, FI-02150 Espoo, Finland
[2]Membrane Transport Discovery Lab, and Department of Biomedical Research, Inselspital, University of Bern, CH-3010 Bern, Switzerland
[*]christopher.landowski@vtt.fi

Supplementary information

Supplementary table S1. Accession numbers and references for the proteins in the phylogenetic tree shown in Fig. 1. Protein IDs for *T. reesei* transporters refer to QM6a genome version 2.0[1]. RUT-C30 genome[2] versions of the protein were used for Tr_CRT1 (TrireRUT-C30_109243), Trire2_67752 (TrireRUT-C30_79984), Trire2_72383 (TrireRUT-C30_133083), Tr_XLT1 (TrireRUT-C30_33630), Trire2_123120 (TrireRUT-C30_26008) and Trire2_4774 (TrireRUT-C30_85251), as indicated in the table. Abbreviations: glc = D-glucose, fru = D-fructose, gal = D-galactose, mann = D-mannose. rha = L-rhamnose, sor = L-sorbose, xyl = D-xylose, ara = L-arabinose, cb = cellobiose, c3 = cellotriose, c4 = cellotetraose, lac = lactose, m2 = mannobiose, x2 = xylobiose, x3 = xylotriose, x3 = xylotetraose, mal = maltose, suc = sucrose, raf = raffinose, galUA = D-galacturonic acid, glcUA = D-glucuronic acid, cbA = cellobionic acid.

Organism	Transporter	Accession	Substrates	Reference
Aspergillus flavus	Afla_ST1	XP_001820343	cb, x2, x3	3
Aspergillus nidulans	Anid_LacpA	Q5B8D1		4
	Anid_LacpB	Q5B9G6	cb, lac	5–7
	Anid_CltA	Q5ATN3	cb	6
	Anid_Xtrd	Q5BGT0	glc, xyl, fru, gal, mann	8
	Anid_MstC/HxtB	Q5AYG1	glc, xyl, fru, gal, mann	6,9–11
	Anid_HxtC	C8VDD8	glc, fru, gal, mann	11
	Anid_MstA/HxtD	Q5ASJ3	glc	9,11
	Anid_HxtE	Q5BCD3	glc, fru, gal, mann	11
	Anid_MstE	AN5860		9
	Anid_ST15	XP_657617	x2, x3	3
Aspergillus niger	Anig_RhtA	XP_025455900	rha, fru	12
	Anig_XltA	XP_026623817	glc, xyl, gal, mann	13
	Anig_XltB	XP_025455052	xyl, mann, cb	13
	Anig_XltC	XP_025450664	glc, xyl, fru, gal, mann	13
	Anig_MstA	A2R055	glc, xyl, fru, mann	14,15
	Anig_MstG	XP_025453682	glc, gal, mann, suc	16
	Anig_MstH	XP_001396930	glc, fru, gal, mann, suc	16

Continued on the next page

Continued from the last page

Organism	Transporter	Accession	Substrates	Reference
	Anig_GatA	A2R3H2	galUA	17,18
	Anig_CtA	A2R0P0	cb, c3, c4, c5	19
	Anig_1186134	XP_001396690	fru, sor	20
	Anig_296054	XP_001391049	fru, sor, mann	20,21
	Anig_mstC	XP_001399490.1	glc, mann, gal	21
	Anig_mstE	XP_001390118	mann, fru, gal	21
	Anig_An01g00850	XP_001388529.2	mann, fru, gal	21
	Anig_An11g09600	A2QXN5	mann	21
	Anig_An12g05820	A2QZQ8	suc	21
	Anig_An02g07610	XP_001399894	suc	21
	Anig_An03g01750	XP_001390077	mann	21
	Anig_An06g00620	XP_001390889	gal	21
Aspergillus oryzae	Ao_MalP	Q96WT5	mal	22
Fusarium graminearum	Fgra_ST2	XP_011322622	cb, x2, x3	3
Fusarium oxysporym	Foxy_ST3	XP_018236089	x2, x3	3
Neurospora crassa	Nc_An25	Q7SEB3	xyl	23
	Nc_CDT-1	Q7SCU1	cb, c3, c4, lac, m2	7,24,25
	Nc_CDT-2	Q7SD12	cb, c3, lac, x2, x3, x4, m2	3,7,24–26
	Nc_GAT-1	Q7SD36	galUA, glcUA	27
	Nc_GLT-1	Q1K4S3	glc, xyl	28,29
	Nc_HGT-1	Q7S0I5	glc	29,30
	Nc_HGT-2	Q7S3Q4	glc	29
	Nc_CBT-1	Q7S555	cba	31,32
	Nc_XYT-1	Q7S5X9	xyl	28
	Nc_XAT-1	F5HC09	xyl, ara	28
	Nc_HXT-1	Q7SHD8	glc, fru, gal, sor, mann	33
	Nc_HXT-2	Q7S496	glc, fru, gal, sor	33
	Nc_NCU00809	Q7SEC5	cb	34
	Nc_LAT-1	Q7S4D2	glc, fru, gal, sor, mann, suc	28,35,36
	Nc_SUT-28	Q7S511		37
	Nc_RCO-3	Q92253		38
Myceliophtora thermophila	Mt_LAT-1	G2QFT5	ara	35
Penicillium chrysogenum	Pc_LacpA/ST	B6H4F0	cb	39,40
	Pc_AraT	B6HE12	ara	41
Penicillium oxalicum	Po_CdtC	EPS25673	cb	42
	Po_CdtD	EPS25817	cb	42
	Po_CdtG	EPS34431	cb, glc, lac	42,43
Phanerochaete sordida	Psor_hxt1	LC438459	glc, xyl, fru, mann	44
Postia placenta	Ppla_ST7	OSX60980	cb, x2, x3	3
Thielavia terrestris	Tter_ST/ST9	XP_003656976	cb	3,40
	Tter_ST13	XP_003650792	cb, x2, x3	3
	Tter_ST17	AEO63901	cb, x2, x3	3
Trichoderma harzianum	Thar_gtt1	Q8TFF4	glc	45
T. reesei	Tr_CRT1[1]	ETS03552	cb, glc, lac	7,40,46
	Tr_HXT1	G0RQV6		47
	TRIRE2_46819	G0RFU7		

Continued on the next page

Continued from the last page

Organism	Transporter	Accession	Substrates	Reference
	Tr_STP1	G0RIJ1	glc, fru, gal, mann, cb, xyl, ara	46,48,49
	TRIRE2_50618	G0RQZ2		
	Tr_STR1	G0RRR0	glc, xyl, fru, gal, mann, ara	13
	TRIRE2_56684	G0RB79		
	Tr_STR3	G0RKF0	glc, xyl, fru, gal, mann	13
	TRIRE2_62502	G0RKB2		
	Tr_XLTR1	G0RN44	glc, mann, fru, xyl	50
	TRIRE2_65191	G0RNV9		
	TRIRE2_67469	G0RSB2		
	TRIRE2_67752[1]	ETS01940	cb	51
	TRIRE2_68812	G0RUI6		
	TRIRE2_69957	G0RW02	xyl, mann, cb	52
	TRIRE2_72383[1]	ETR98700		
	TRIRE2_76800	G0RFZ0		
	TRIRE2_77517	G0RIF6		53
	TRIRE2_79202	G0RN18		53
	TRIRE2_82309	G0RWI3		
	Tr_XLT1[1]	ETS04871	xyl	54
	TRIRE2_106330	G0RHS6	galUA	18
	TRIRE2_106556	G0RGR7		
	Tr_STR2	G0RH09	glc, xyl, fru, gal, mann	13
	TRIRE2_121850	G0RJM5		
	TRIRE2_4774[1]	ETR99734		
	TRIRE2_48444	G0RJZ0		
	TRIRE2_55077	G0RA72		
	TRIRE2_60945	G0RIF7		
	TRIRE2_69026	G0RUM8	galUA	18
	TRIRE2_69901	G0RW16		
	TRIRE2_105260	G0RF04		
	TRIRE2_59388	G0RFJ2		
	TRIRE2_109677	G0RQ49		
	TRIRE2_122013	G0RJY9		
	TRIRE2_123809	G0RUN3		
	TRIRE2_26160	G0RI84		
	TRIRE2_27770	G0RRJ5		
	TRIRE2_27939	G0RUM4		
	TRIRE2_53903	G0R951		
	TRIRE2_5656	G0RUS5		
	TRIRE2_65153	G0RP99		
	TRIRE2_65493	G0RPR8		
	TRIRE2_67541	G0RSV0		
	TRIRE2_69651	G0RVI9		
	TRIRE2_76758	G0RFS1		
	TRIRE2_77785	G0RJ14		
	TRIRE2_80091	G0RQ60		
Trichoderma virens	Tv_ST16	XP_013958763	x2	3

Continued on the next page

Continued from the last page

Organism	Transporter	Accession	Substrates	Reference
	Tv_SUT	XP_013949637	suc	55
Ustilago maydis	Um_Srt1	A0A0D1E1N5	suc	56,57
	Um_Hxt1	A0A0D1DTL9	glc, fru, gal, mann	58
Amanita muscaria	Am_MST1	O13411	glc, fru	59
Botrytis cinerea	Bc_FRT1	Q5XTQ5	glc, fru	60
Colletotrichum graminicola	Cg_Hxt1	D2TG02	glc, fru, mann	61
	Cg_Hxt2	D2TG03	glc, fru, gal, mann	61
	Cg_Hxt3	D2TG04	glc, fru, gal, mann	61
	Cg_Hxt5	D2TG06	glc, fru, gal, mann	61
	Cg_Mbt1	D2TG08	mal, raff	62
Uromyces fabae	Uf_HXT1	Q96VF4	glc, fru	63
Puccinia striiformis	Ps_HXT1	A0A0L0VC49	glc, fru, mann, xyl, ara, suc	64
Glomus sp.	Gs_MST2	G3CES2	glc	65
Geosiphon pyriformis	Gp_MST1	A0ZXK5	glc, gal, mann	66

[1]RUT-C30 version was used.

Supplementary table S2. K_m values (in mM) of monosaccharide transporters from filamentous fungi. H^+ indicates sugar/H^+ symport activity. Values are in mM. Uncertainties were omitted for clarity. Abbreviations as in Supplementary table S1. nd = not determined.

**Organism**	**Protein**	H^+	**glc**	**mann**	**gal**	**fru**	**xyl**	**ara**	**Reference**
A. muscaria	MST1	x	0.46			4.2			59
A. nidulans	hxtB	x	15				0.54		6,10,11
A. niger	mstA	x	0.025	0.06		4.5	0.3		15
	mstG	x	0.5						16
	mstH	x	0.06						16
	xltA	nd	0.07				0.09		13
	xltB	nd					14		13
	xltC	nd	0.11				4.71		13
	1186134	x				0.066			20
	296054	x				0.1			20
B. cinerea	FRT1	x				1.1			60
C. graminicola	Hxt1	x	0.0206						61
	Hxt2	x	0.136						61
	Hxt3	x	0.013						61
	Hxt5	x	0.92						61
G. pyriformis	MST1	x	1.2						66
Glomus sp.	MST2	x	0.033						65
M. thermophila	LAT-1	x						29.39	35
N. crassa	An25	-					175.7		23
	HGT-1	nd	0.01613						29
	HGT-2	nd	0.09897						29
	GLT1	nd	18.42						29
	XAT-1	nd					18.17	61.93	28
	XYT-1	nd					7.58		28
	LAT-1	x						58.12	35
P. chrysogenum	AraT	x						0.13	41
P. striiformis	HXT1	x	0.059						64
T. reesei	STP1	-	0.00738						This study, 49
	STR1	nd	0.01				5.7		13
	STR1	x	0.1461				0.0411	5.79	This study
	STR2	nd	0.05				6.18		13
	STR3	nd	0.06				2.19		13
	STR3	x	0.0579	0.1862	1.11		2.74		This study
	FRT1	x				13.95			This study
	MLT1	x	39.74						This study
	GLT1	-	3.04						This study
U. fabae	HXT1	x	0.36			1			63
U. maydis	Hxt1	x	0.018						58

Supplementary table S3. K_m (in mM) values of di-/trisaccharide and sugar acid transporters from filamentous fungi. H^+ indicates sugar/H^+ symport activity. Values are in mM. Uncertainties were omitted for clarity. Abbreviations as in Supplementary table S1. nd = not determined.

Organism	Protein	H^+	glc	CB	c3	lac	galUA	glcUA	suc	mal	raf	Reference
A. niger	GatA	nd					0.34					18
C. graminicola	Mbt1	-								8.7	0.31	62
N. crassa	CDT-1	x		0.004								24,67
	CDT-1	x		0.0044		0.0388						7
	CDT-1	x		0.0304	0.143	0.0454						This study
	CDT-2	-		0.0032								24,67
	GAT-1	x					0.0012					27
T. reesei	CRT1	x		0.0031		0.0629						7
	CRT1	x	9.26	0.0499		0.0831						This study
	GAT1	x					0.155	0.008				This study
	GAT2	x					0.015	0.031				This study
T. virens	SUT								1.5			55
U. maydis	Srt1	x							0.026			56
	Srt1[1]								0.09			57
	Srt1[2]								0.12			57

[1]Determined at pH 5.5
[2]Determined at pH 6.5

Supplementary table S4. Primers used in this study

Name	Sequence
PP-203	CGCGGATCCATGGCCATCGCCATG
PP-204	CCCAAGCTTTCAGTAACTGAAGTCAACG
PP-209	ATGCGGATCCATGGCTCCCTCTGCGGCGC
PP-210	ATGCGAATTCTTATACAGATGAACCGGAAACC
PP-211	ATGCTCTAGAATGTACTTGAAGCTCATCAGCG
PP-212	ATGCAAGCTTCTACTCTTGATGCAGCTCGGC
SaSS-19	GGTCAATGCAAGAAATACATATTTGGTCT
SaSS-20	CATTCGTGACTATAAAATGAATAAACTAAACTATTC
SaSS-57	ATTCGGGTGTTCTTGAGGCTGG
SaSS-75	AAATCTATAACTACAAAAAACACATACAGGACAATGCGCTTCTTCAAGAACTATC
SaSS-76	TCAGTTAGCTAGCTGAGCTCGACTCTAGAGTTATACAGCCTTGTCGGCAGG
SaSS-95	TACAAAAGGATCCATGCGCTTCTTCAAGAACTATC
SaSS-96	CATGATCGAATTCTTATACAGCCTTGTCGGCAG
TPI1_P_long	GAAATTAATTAAAGCAATC
TPI1_T_long	GTATCGGTCAGTCATTAATAC

Supplementary table S5. Plasmids used in this study. The MoClo-based expression vector for yeast expression contained *PGK1* promoter, *ENO1* terminator and *CEN6/ARS4* origin of replication for low copy expression. QM6a version of the *T. reesei* genes was used unless otherwise specified. The manually annotated sequences are presented in Supplementary Fig. S10.

Name	Description	Reference
S. cerevisiae expression		
B2159	P_{TPI1}, T_{TPI1}, 2 µm, amp^R	68
pSS17	Trire2_72383 (QM6a version) in MoClo-based expression vector	This study
pSS27	Trire2_72383 (RUT-C30 version) in MoClo-based expression vector	This study
pSS35	STR3/Trire2_62380 (RUT-C30 version) in MoClo-based expression vector	This study
pSS36	Trire2_106556 in MoClo-based expression vector	This study
pSS14	Trire2_47710 in MoClo-based expression vector	This study
pSS40	Trire2_62502 in MoClo-based expression vector	This study
pSS16	Trire2_50894 in MoClo-based expression vector	This study
pSS29	Trire2_77517 in MoClo-based expression vector	This study
pSS51	Trire2_56684 (QM6a version) in MoClo-based expression vector	This study
pSS30	Trire2_56684 (manually annotated) in MoClo-based expression vector	This study
pSS18	Trire2_79202 in MoClo-based expression vector	This study
pSS32	Trire2_67469 (manually annotated) in MoClo-based expression vector	This study
pSS31	Trire2_65191 in MoClo-based expression vector	This study
pSS87	Trire2_69026 in B2159	This study
pSS49	CEL1a/Trire2_120749 in MoClo-based expression vector	This study
X. laevis expression		
pol1	P_{T7}, *X. laevis* β-globin 5' and 3' UTR, poly(A) tract, Amp^R	69
pMJB08	pol1 with additional tags and modified multiple cloning site	70
pSS79	pol1 modified to be compatible with the MoClo system	Havukainen *et al.*, submitted
B7287	Trire2_72383 (QM6a version) in pMJB08	This study
pSS81	STR3/Trire2_62380 (RUT-C30 version) in pSS79	This study
pSS106	Trire2_50618 in pSS79	This study
pSS80	Trire2_106556 in pSS79	This study
pSS108	Trire2_62502 in pSS79	This study
pSS107	STR1/Trire2_50894 in pSS79	This study
B7290	Trire2_77517 in pMJB08	This study
pSS82	Trire2_67752 (RUT-C30 version) in pSS79	This study
pSS110	Trire2_56684 (manually annotated) in pSS79	This study
pSS83	CRT1/Trire2_3405 (RUT-C30 version) in pSS79	This study
B7291	Trire2_79202 (manually annotated) in pMJB08	This study
pSS113	Trire2_69957 in pSS79	This study
pSS111	Trire2_67469 (manually annotated) in pSS79	This study
pSS109	Trire2_65191 in pSS79	This study
pSS114	Trire2_69026 in pol1	This study
pSS84	Trire2_106330 in pSS79	This study
pSS104	CDT-1/NCU00801 in pSS79	This study
pSS105	CDT-2/NCU08114 in pSS79	This study

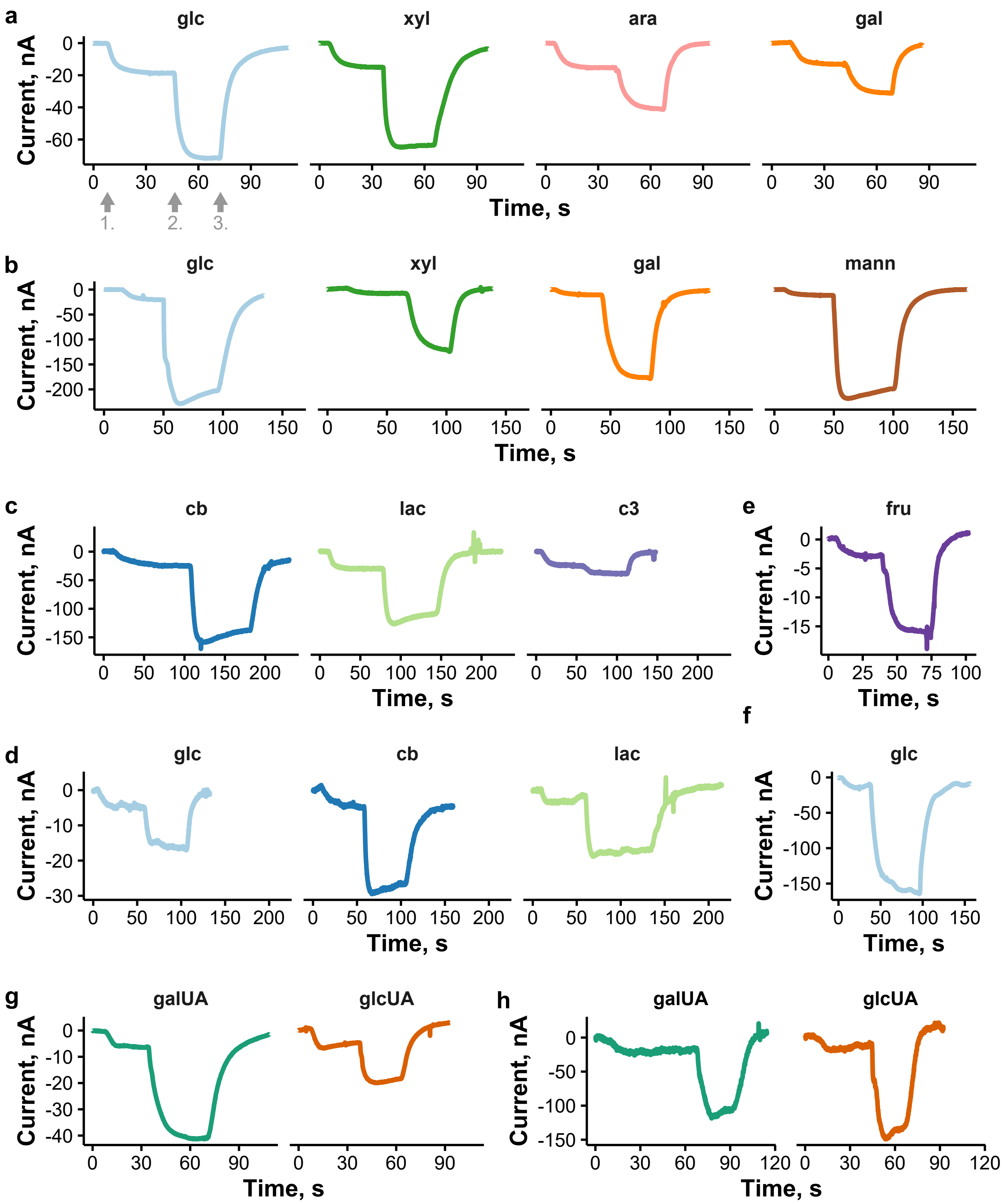

Supplementary figure S1. Current trace recordings of the identified symporters. (**a**–**h**) Traces of STR1 (**a**), STR3 (**b**), CDT-1 (**c**), CRT1 (**d**), Trire2_50618 (FRT1) (**e**), Trire2_67469 (MLT1) (**f**), Trire2_106330 (GAT1) (**g**) and Trire2_69026 (GAT2) (**h**) for selected sugars. Arrows in panel **a** indicate the timeline of the experiment: 1. perfusion with ND-96 (pH 5.5), 2. perfusion with the same buffer with sugar, 3. perfusion with ND-96 (pH 7.4) to return to baseline. Same steps can be identified in each subpanel. The oocytes were clamped at -50 mV and the sugars were in 5 mM concentration except for cellotriose (c3) in panel **c** and D-glucose in panel **f**, which were in 10 µM and 200 mM concentrations, respectively. Abbreviations as in Supplementary table S1. The figure was created with `ggplot2` package for R[71,72].

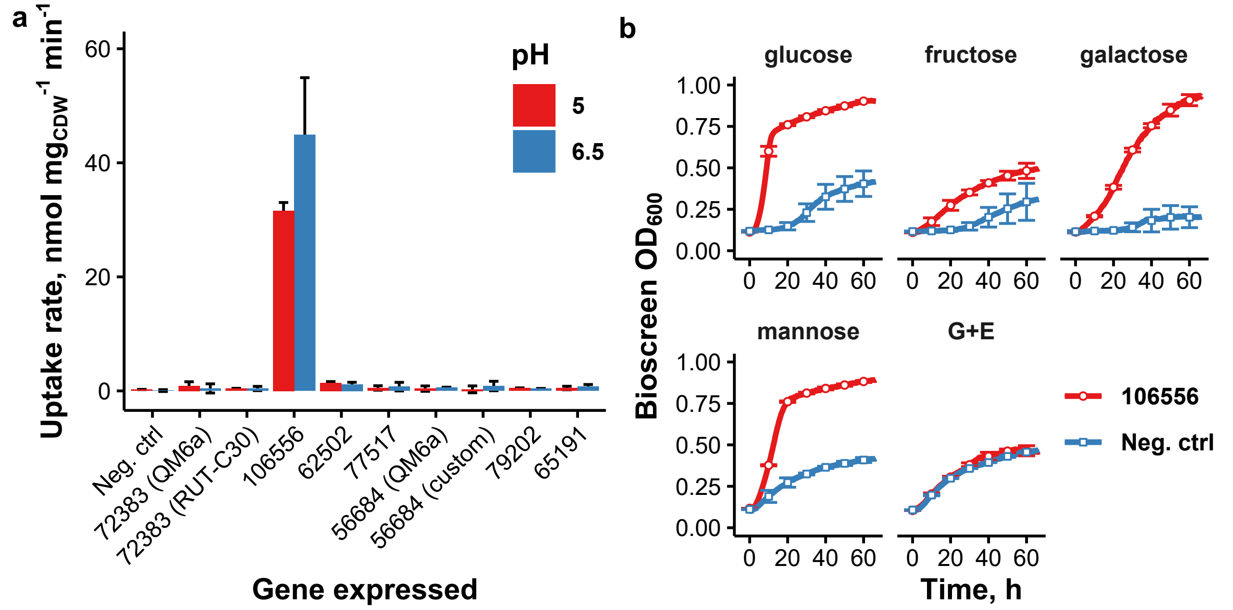

Supplementary figure S2. Identification of transport activity of Trire2_106556 (GLT1). (**a**) Representative experiment showing D-glucose uptake rates of uncharacterized *T. reesei* transporters which did not function as symporters in oocytes. D-glucose concentration was 5 mM and the assay was done in two different pH values. Error bars present standard deviation from two technical replicates. (**b**) Representative experiment showing growth curves of strain expressing Trire2_106556 and negative control strain at various monosaccharide carbon sources. The carbon sources were at 0.5% concentration, except for glycerol and ethanol (G+E), which were at 2% concentration. Error bars present standard deviation between 3 technical replicates, and they and points are shown only for every 10 h for clarity. The figure was created with `ggplot2` package for R[71,72].

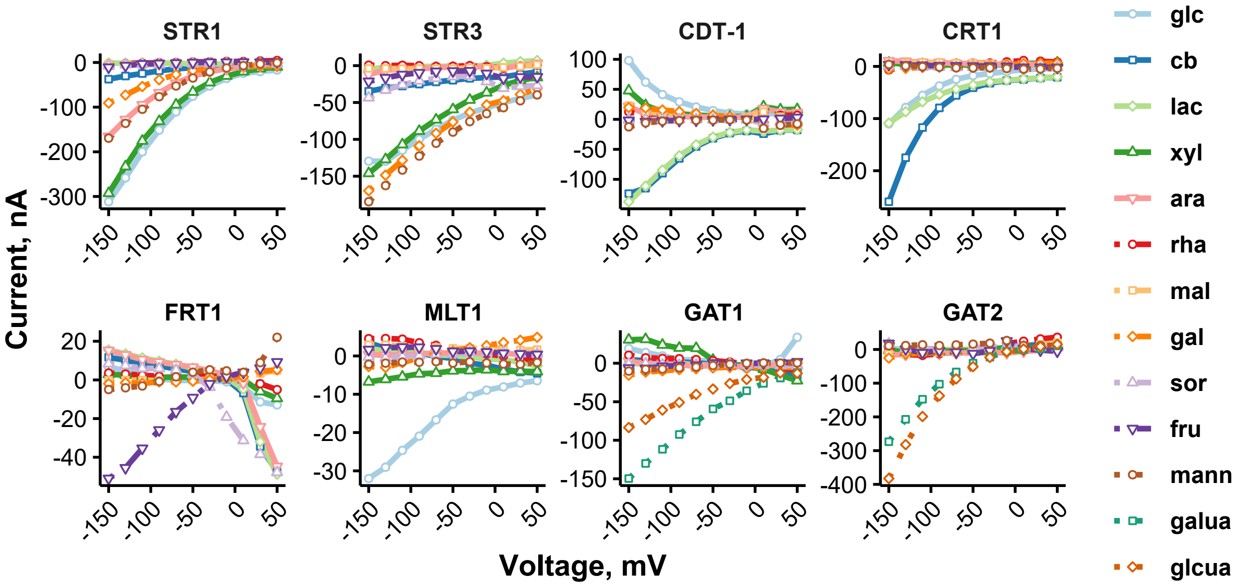

Supplementary figure S3. Representative I-V curves for each of the identified symporters. Sugars were at 5 mM concentration. Abbreviations as in Supplementary table S1. The figure was created with `ggplot2` package for R[71,72].

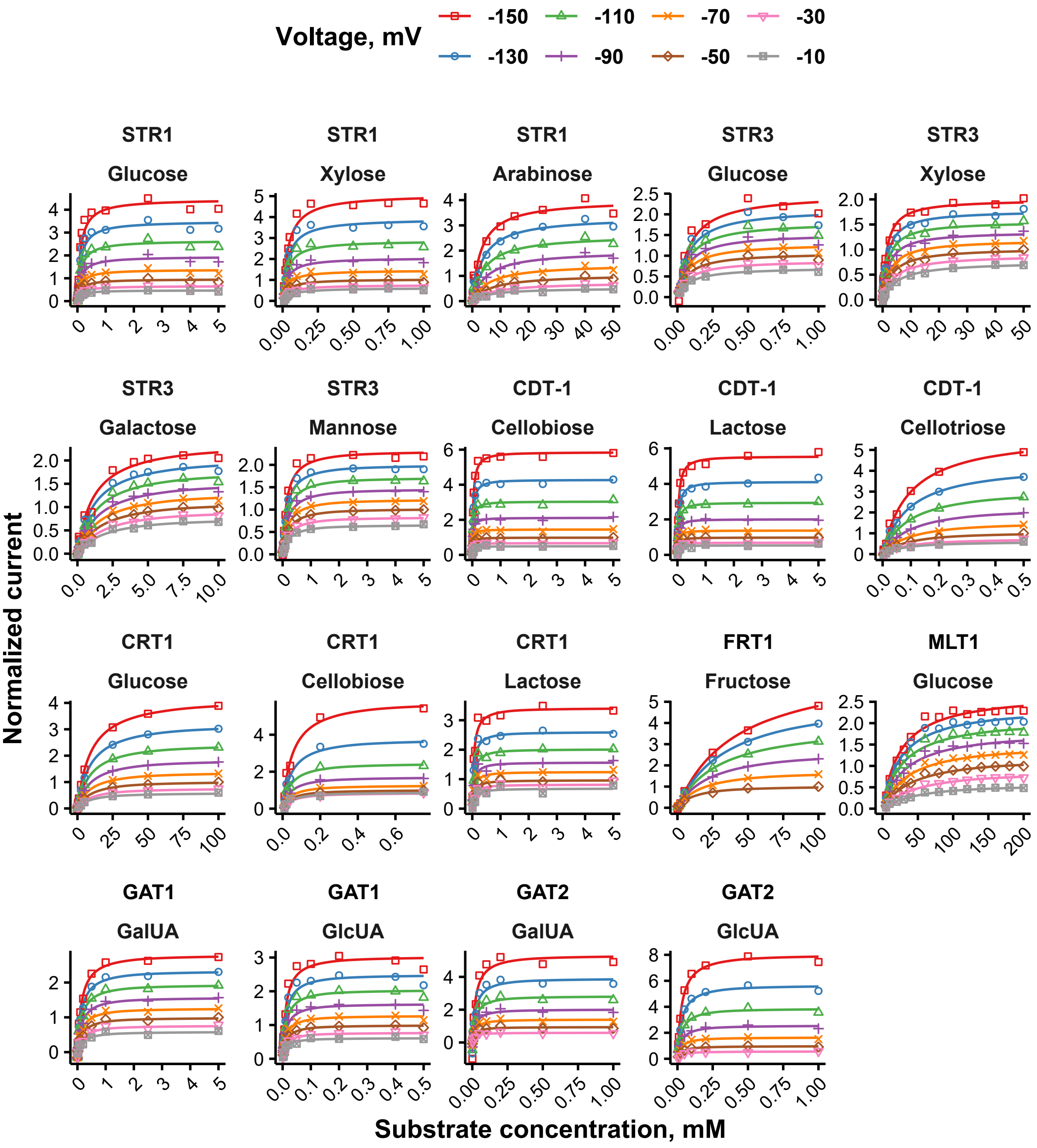

Supplementary figure S4. Substrate-induced currents at different voltages for sugar and sugar acid substrates of the identified symporters. Currents were normalized to the highest value at -50 mV. Values obtained at voltages below 0 mV are shown, expect for Trire2_50618 (FRT1) and Trire2_69026 (GAT2), for which values obtained below -30 mV and -10 mV are shown, respectively. Representative experiments are shown. The figure was created with `ggplot2` package for R[71,72].

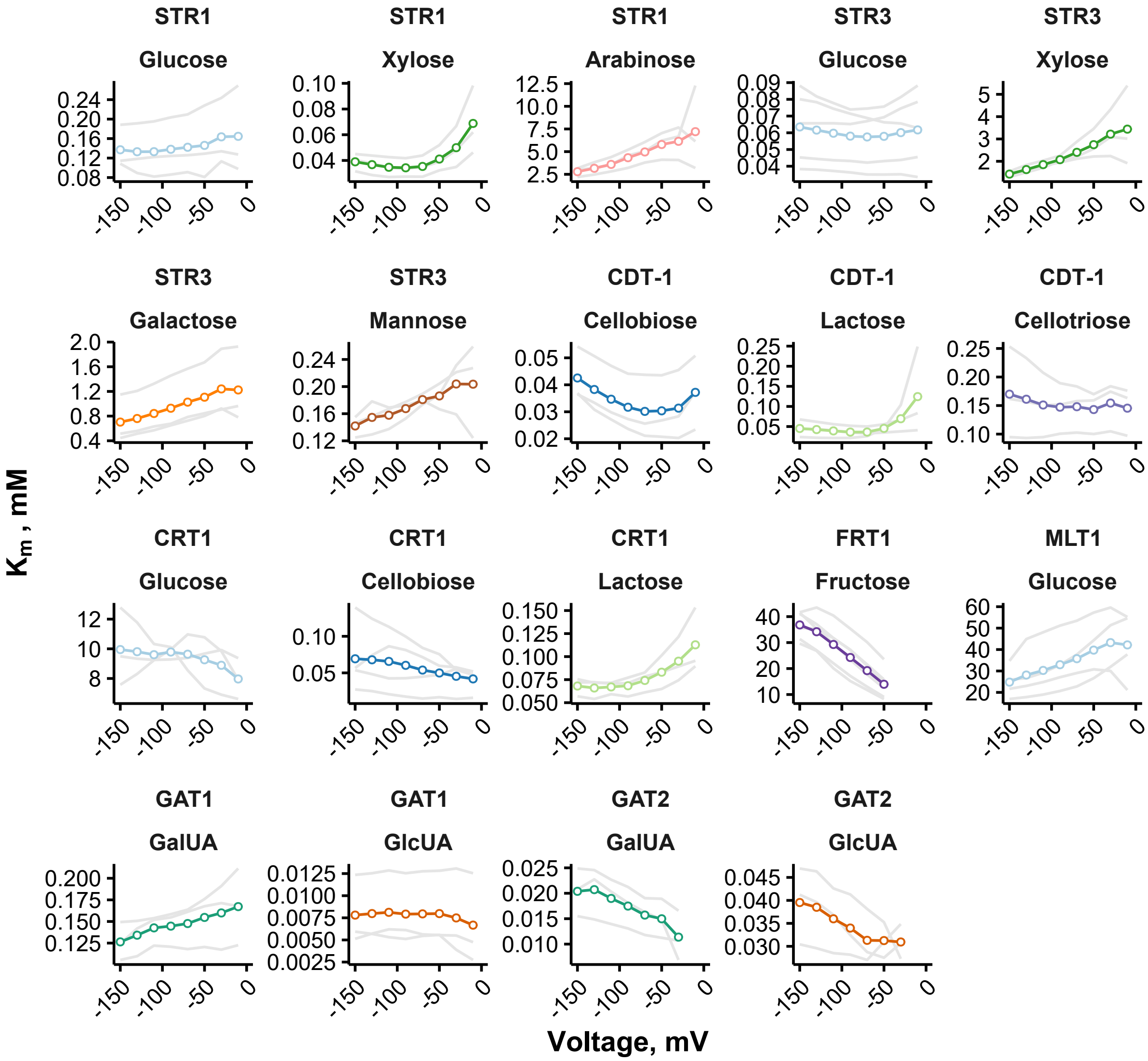

Supplementary figure S5. Voltage-dependence of K_m values. Colored lines and points present average K_m value across experiments. Light gray lines present results from individual experiments ($n \geq 3$). Values obtained at voltages below 0 mV are shown, expect for Trire2_50618 (FRT1) and Trire2_69026 (GAT2), for which values obtained below -30 mV and -10 mV are shown, respectively. The figure was created with `ggplot2` package for R[71,72].

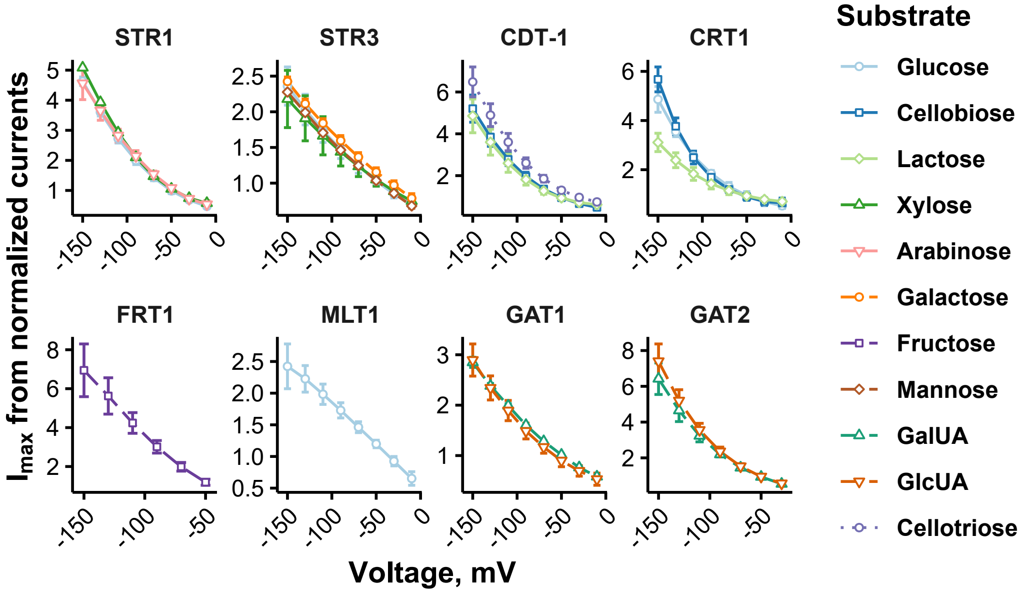

Supplementary figure S6. Voltage-dependence of I_{max} values for each transporter. I_{max} values were calculated from currents normalized to highest value at -50 mV (see Supplementary Fig. S4). Values obtained at voltages below 0 mV are shown, expect for Trire2_50618 (FRT1) and Trire2_69026 (GAT2), for which values obtained below -30 mV and -10 mV are shown, respectively. Points and error bars present the mean and standard deviation between individual experiments ($n \geq 3$). The figure was created with `ggplot2` package for R[71,72].

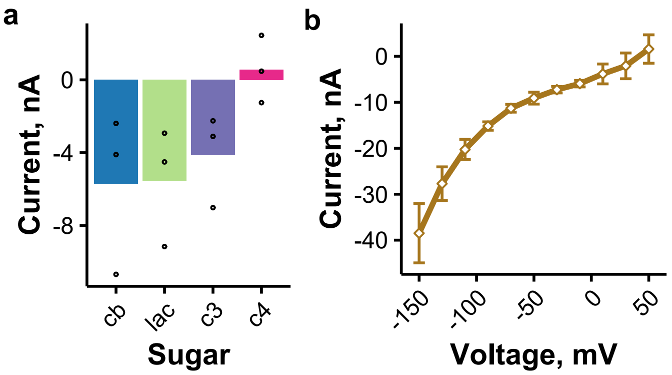

Supplementary figure S7. Further selectivity testing with CDT-1 and CRT1. (**a.**) Selectivity plots of CDT-1 with cellobiose, lactose, cellotriose or cellotetraose in 10 µM concentration at -50 mV. Points present results from individual experiments and bars their average ($n = 3$). Abbreviations as in Supplementary table S1. (**b.**) Current traces for CRT1 with 1 mM α-sophorose. Points and error bars present mean and standard deviation between oocytes ($n = 3$). The figure was created with `ggplot2` package for R[71,72].

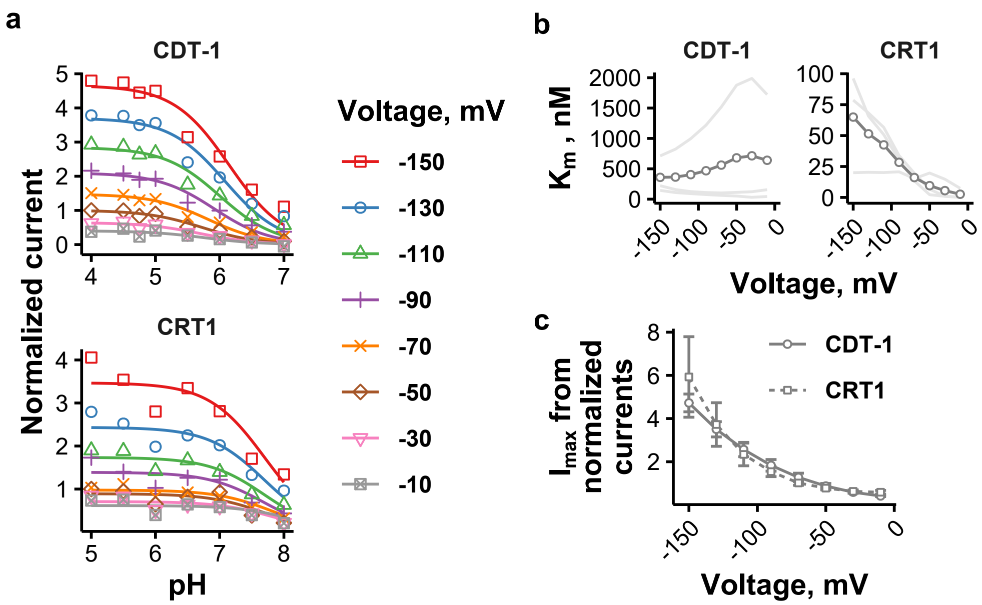

Supplementary figure S8. Voltage-dependence of pH-dependence kinetics. (**a.**) Currents induced by 5 mM cellobiose at different voltages as a function of pH. Representative experiments are shown. (**b.**) Voltage-dependence of K_m value for protons. Points and dark lines present average K_m values across experiments, while light gray lines present results from individual experiments ($n = 3$). Voltages below 0 mV are shown. (**c.**) Voltage-dependence of I_{max} for protons. Points present average I_{max} values calculated from currents normalized to highest value at -50 mV (see panel **a**), while error bars present their standard deviation ($n = 3$). Voltages below 0 mV are shown. The figure was created with `ggplot2` package for R [71,72].

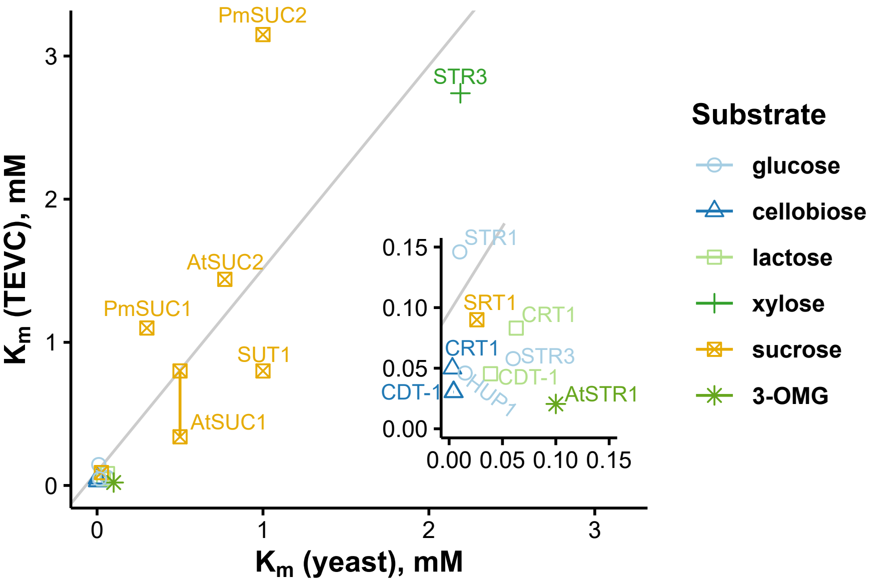

Supplementary figure S9. Relationship between K_m values obtained from yeast and *X. laevis* oocytes with TEVC. Points present values for independent transporters and the gray line represents linear fit for the data ($R^2 = 0.7269$). Two TEVC K_m values were available for AtSUC1, and thus the points were joined with colored line. Inset shows transporters with K_m below 0.15 mM. Error bars were omitted for clarity. Data was available for the following transporters: *T. reesei* CRT1[7], STR1[13] and STR3[13]; *N. crassa* CDT-1[7,24]; *U. maydis* SRT1[56,57]; *Solanum tuberosum* SUT1[73,74]; *Arabidopsis thaliana* STP1[75,76] SUC1[77–79] and SUC2[77,80]; *Chlorella kessleri* HUP1[81,82]; *Plantago major* SUC1[79,83] and SUC2[79,84]. *T. reesei* STR1 K_m for D-xylose was excluded from the analysis (see Results and Discussion). K_m values obtained at -50 mV were used for transporters analyzed in this study (CRT1, STR1, STR3 and CDT-1) and for HUP1, whereas the K_m values for UmSRT1 and AtSUC2 were obtained at -130 and -137 mV, respectively, and those of *A. thaliana* and *P. major* SUC1 and SUC2 were the average of values obtained at the smallest and highest test voltages[57,78–80,82]. Abbreviations: At = *A. thaliana*, Pm = *P. major*, 3-OMG = 3-O-methyl-D-glucose. The figure was created with `ggplot2` package for R[71,72].

a **MASSGIEKE**MASADGVDIQSSKDVTTGGVQDVDGGAGDFSHDQRLVIESARAAAAKEQSMTLLQGIKLYPKAIAWSI
LISTCIVMEGYDVSLVNNFYAFPQFNEKYGELYPDGTYQVPARWQSGLSNGATVGEIIGLFINGFVSERFGYRKTVM
GCLILVAAFTAIFFTAPNVETLLVAEILCGIPWGIFQTITVTYASEVCPVALRGYLTTYVNFCWGLGQEIGIGVIYA
MLKRNDEWAYRIPYGLQWMWPLPLFIAIYFAPESPWWLVRQGKAEEAKKSLLRLTSLNRETDFDADETVAMMVHTTA
LEEKMTAGATYWDCFKGVDLRRTEIVCMTWAIQNLSGNAFSNYSTYFLQQAGLSTKDSYSFALGQYAINMVGVFGAW
GLMTMGVGRRSLYLYGLCGLCSMLFILGFLGLVPESHRREGSLATGSIMVVWALFYQLTVGTVCYSLVGELSSRRLQ
IKTVVLARNLYNIVGIVTNVLTPYMLNPKAWDWSNYTGFFWGGICFCCIVYTYFRLPEPRGRTFAELDVLFEKKIPA
RKFSSTKVDVFHESIDEKVMHQYDGIMKTHVEKISFV

c **MDSSKVTDHDIVQDVVHDESPVKVTIPNCMTASR**MQAALLRENPHIFRNSFLKLYGCIFVGYLCSATNGFDSNTFGG
LSAIPQFTRYFGITTQNQGLVAALYVIGNIAGSFVAGPCSDTYGRRVGMAVGSAICVIGAVLQTASTTLSMLMGGRF
ILGMGAVLVQTAGPSYVVEMSYPKYRAQLTGAYQACFFLGTIVSTWLEFGLNHAKTTLSYPWRLPLAIQGLPSVMIL
CAVWFIPESPRWCVQHGRVDEAKAILIKYHGDGDPDALVANLELQEMIEVIQMDGSDKRWWDYRCLFDTRAARYRTF
LVLCIAWFGELDLPPTSYYFPLMVKTVGITDVNTQLLLNAIQTPVMAVSALCGLSVVHKFGRRKLLMFSSAGMSVSI
AIITACTALQAGRPAVGGTGIAFLYIFLIVFAFAWTPMQSLYPVEVLSFNSRAKGMAFLAFMNNAVKVMNTYVPPIA
IANSGWKYYFLYVFWDAFGVIVIYFFFVETRDWSLEEIEDLFQAKNPVKASLEKKRISVAYDGTIAHVPDGRDDV

b MYLKLISAGFSFFVAGVNDGAIGALIPYFIRDYNVTTAIVSSVYGANFLGWLFAAITNTHLRQYLDLGAMLALGAAF
QIAAHALRSWEPPFGLFVVTFWLVSVGQAFQDTHANSWVASAVPKGAHRWLAFIHAMYMAGCLVGPFVSTAVASAGE
VSRWYLFYTFPLGLGVMNLVLTCVAFRDTLGLQRKSSASE**SQGRTLGETAAAEPSNEAV**SRNKEATQLIKKTASTPS
VWLLSLFFFFYLGSVLTAGGWVVEYLVNVRHGNLSQMGYVPAGFNGGGLLGRLLLAEPTHRFGERRMVFLYVVASIG
LQLIFWLVPNIVAASIAVSLIGFFTGPLFPTGISLGSKLFPSDIHSTALPLVFVFAQLGGSLFPIITGVVSADAGVK
VLQPMLIALLTVTAISWLLVPSPKTSNAELHQE

Supplementary figure S10. Manually annotated *T. reesei* transporter sequences used in this study. Residues which are not present in the QM6a annotation are in bold. (**a**) Amino acid sequence of Trire2_67469 (MLT1) was extended based on the sequence of putative maltose permease from *T. parareesei* (GenBank OTA04610.1). The part that is missing from the QM6a gene corresponds to nucleotides 61,612–61,586 in scaffold 21 (minus-strand), and is adjacent to the start codon of the QM6a gene. (**b**) Amino acid sequence of Trire2_56684 was extended based on the sequence of MFS permease from *T. guizhouense* (OPB43417). The part that is missing from the QM6a gene corresponds to nucleotides 62,906–63,007 (plus-strand), and is adjacent to the start codon of the QM6a gene. (**c**) Amino acid sequence of Trire2_79202 that was obtained from cDNA. The insertion corresponds to the absence of the second intron from the QM6a sequence. The figure was created with Inkscape software (version 1.01, https://inkscape.org/).

References

1. Martinez, D. *et al.* Genome sequencing and analysis of the biomass-degrading fungus *Trichoderma reesei* (syn. *Hypocrea jecorina*). *Nat. biotechnology* **26**, 553 (2008).

2. Jourdier, E. *et al.* Proximity ligation scaffolding and comparison of two *Trichoderma reesei* strains genomes. *Biotechnol. Biofuels* **10**, 151 (2017).

3. Zhang, C., Acosta-Sampson, L., Yu, V. Y. & Cate, J. H. Screening of transporters to improve xylodextrin utilization in the yeast *Saccharomyces cerevisiae*. *PloS one* **12**, e0184730 (2017).

4. Fekete, E. *et al.* Identification of a permease gene involved in lactose utilisation in *Aspergillus nidulans*. *Fungal Genet. Biol.* **49**, 415–425 (2012).

5. Fekete, E. *et al.* Characterization of a second physiologically relevant lactose permease gene (*lacpB*) in *Aspergillus nidulans*. *Microbiology* **162**, 837–847 (2016).

6. Reis, T. F. *et al.* Identification and characterization of putative xylose and cellobiose transporters in *Aspergillus nidulans*. *Biotechnol. Biofuels* **9**, 204 (2016).

7. Havukainen, S., Valkonen, M., Koivuranta, K. & Landowski, C. P. Studies on sugar transporter CRT1 reveal new characteristics that are critical for cellulase induction in *Trichoderma reesei*. *Biotechnol. Biofuels* **13**, 1–20 (2020).

8. Colabardini, A. C. *et al.* Functional characterization of a xylose transporter in *Aspergillus nidulans*. *Biotechnol. Biofuels* **7**, 46 (2014).

9. Forment, J. V. *et al.* High-affinity glucose transport in *Aspergillus nidulans* is mediated by the products of two related but differentially expressed genes. *PLoS One* **9**, e94662 (2014).

10. Dos Reis, T. F. *et al.* The low affinity glucose transporter HxtB is also involved in glucose signalling and metabolism in *Aspergillus nidulans*. *Sci. Rep.* **7**, 45073 (2017).

11. Dos Reis, T. F. *et al.* Identification of glucose transporters in *Aspergillus nidulans*. *PLoS One* **8**, e81412 (2013).

12. Sloothaak, J., Odoni, D. I., Martins dos Santos, V. A., Schaap, P. J. & Tamayo-Ramos, J. A. Identification of a novel L-rhamnose uptake transporter in the filamentous fungus *Aspergillus niger*. *PLoS genetics* **12**, e1006468 (2016).

13. Sloothaak, J. *et al.* Identification and functional characterization of novel xylose transporters from the cell factories *Aspergillus niger* and *Trichoderma reesei*. *Biotechnol. Biofuels* **9**, 148 (2016).

14. Jørgensen, T. R. *et al.* Glucose uptake and growth of glucose-limited chemostat cultures of *Aspergillus niger* and a disruptant lacking MstA, a high-affinity glucose transporter. *Microbiology* **153**, 1963–1973 (2007).

15. Vankuyk, P. A. *et al.* *Aspergillus niger mstA* encodes a high-affinity sugar/H^+ symporter which is regulated in response to extracellular pH. *Biochem. J.* **379**, 375–383 (2004).

16. Sloothaak, J. *et al.* *Aspergillus niger* membrane-associated proteome analysis for the identification of glucose transporters. *Biotechnol. Biofuels* **8**, 150 (2015).

17. Sloothaak, J., Schilders, M., Schaap, P. J. & de Graaff, L. H. Overexpression of the *Aspergillus niger* GatA transporter leads to preferential use of D-galacturonic acid over D-xylose. *AMB Express* **4**, 66 (2014).

18. Protzko, R. J. *et al.* Engineering *Saccharomyces cerevisiae* for co-utilization of D-galacturonic acid and D-glucose from citrus peel waste. *Nat. commun.* **9**, 1–10 (2018).

19. Lin, H. *et al.* Identification and characterization of a cellodextrin transporter in *Aspergillus niger*. *Front. Microbiol.* **11**, 145 (2020).

20. Coelho, M. A., Gonçalves, C., Sampaio, J. P. & Gonçalves, P. Extensive intra-kingdom horizontal gene transfer converging on a fungal fructose transporter gene. *PLoS Genet.* **9**, e1003587 (2013).

21. de Vries, R. P. *et al.* Comparative genomics reveals high biological diversity and specific adaptations in the industrially and medically important fungal genus *Aspergillus*. *Genome Biol.* **18**, 1–45 (2017).

22. Hasegawa, S., Takizawa, M., Suyama, H., Shintani, T. & Gomi, K. Characterization and expression analysis of a maltose-utilizing (*MAL*) cluster in *Aspergillus oryzae*. *Fungal Genet. Biol.* **47**, 1–9 (2010).

23. Du, J., Li, S. & Zhao, H. Discovery and characterization of novel D-xylose-specific transporters from *Neurospora crassa* and *Pichia stipitis*. *Mol. BioSystems* **6**, 2150–2156 (2010).

24. Galazka, J. M. *et al.* Cellodextrin transport in yeast for improved biofuel production. *Science* **330**, 84–86 (2010).

25. Hassan, L. *et al.* Crosstalk of cellulose and mannan perception pathways leads to inhibition of cellulase production in several filamentous fungi. *MBio* **10** (2019).

26. Li, X. *et al.* Expanding xylose metabolism in yeast for plant cell wall conversion to biofuels. *Elife* **4**, e05896 (2015).

27. Benz, J. P. *et al.* Identification and characterization of a galacturonic acid transporter from *Neurospora crassa* and its application for *Saccharomyces cerevisiae* fermentation processes. *Biotechnol. Biofuels* **7**, 20 (2014).

28. Li, J., Lin, L., Li, H., Tian, C. & Ma, Y. Transcriptional comparison of the filamentous fungus *Neurospora crassa* growing on three major monosaccharides D-glucose, D-xylose and L-arabinose. *Biotechnol. Biofuels* **7**, 31 (2014).

29. Wang, B. *et al.* Identification and characterization of the glucose dual-affinity transport system in *Neurospora crassa*: pleiotropic roles in nutrient transport, signaling, and carbon catabolite repression. *Biotechnol. Biofuels* **10**, 17 (2017).

30. Xie, X. *et al.* Transcriptional response to glucose starvation and functional analysis of a glucose transporter of *Neurospora crassa*. *Fungal Genet. Biol.* **41**, 1104–1119 (2004).

31. Xiong, Y. *et al.* The proteome and phosphoproteome of *Neurospora crassa* in response to cellulose, sucrose and carbon starvation. *Fungal Genet. Biol.* **72**, 21–33 (2014).

32. Li, X. *et al.* Cellobionic acid utilization: from *Neurospora crassa* to *Saccharomyces cerevisiae*. *Biotechnol. Biofuels* **8**, 1–9 (2015).

33. Gao, J., Wang, B., Han, X. & Tian, C. Genome-wide screening of predicted sugar transporters in *Neurospora crassa* and the application in hexose fermentation by *Saccharomyces cerevisiae*. *Sheng wu Gong Cheng xue bao= Chin. J. Biotechnol.* **33**, 79–89 (2017).

34. Lian, J., Li, Y., HamediRad, M. & Zhao, H. Directed evolution of a cellodextrin transporter for improved biofuel production under anaerobic conditions in *Saccharomyces cerevisiae*. *Biotechnol. Bioeng.* **111**, 1521–1531 (2014).

35. Li, J. *et al.* Functional analysis of two L-arabinose transporters from filamentous fungi reveals promising characteristics for improved pentose utilization in *Saccharomyces cerevisiae*. *Appl. Environ. Microbiol.* **81**, 4062–4070 (2015).

36. Benz, J. P. *et al.* A comparative systems analysis of polysaccharide-elicited responses in *Neurospora crassa* reveals carbon source-specific cellular adaptations. *Mol. Microbiol.* **91**, 275–299 (2014).

37. Wu, V. W. *et al.* The regulatory and transcriptional landscape associated with carbon utilization in a filamentous fungus. *Proc. Natl. Acad. Sci. U.S.A* **117**, 6003–6013 (2020).

38. Madi, L., McBride, S. A., Bailey, L. A. & Ebbole, D. J. *rco-3*, a gene involved in glucose transport and conidiation in *Neurospora crassa*. *Genetics* **146**, 499–508 (1997).

39. Jónás, Á. *et al.* Extra- and intracellular lactose catabolism in *Penicillium chrysogenum*: phylogenetic and expression analysis of the putative permease and hydrolase genes. *The J. antibiotics* **67**, 489 (2014).

40. Bae, Y.-H., Kang, K.-H., Jin, Y.-S. & Seo, J.-H. Molecular cloning and expression of fungal cellobiose transporters and β-glucosidases conferring efficient cellobiose fermentation in *Saccharomyces cerevisiae*. *J. biotechnology* **169**, 34–41 (2014).

41. Bracher, J. M. *et al.* The *Penicillium chrysogenum* transporter *Pc*AraT enables high-affinity, glucose-insensitive L-arabinose transport in *Saccharomyces cerevisiae*. *Biotechnol. Biofuels* **11**, 63 (2018).

42. Li, J. *et al.* Cellodextrin transporters play important roles in cellulase induction in the cellulolytic fungus *Penicillium oxalicum*. *Appl. Microbiol. Biotechnol.* **97**, 10479–10488 (2013).

43. de Ruijter, J. C., Igarashi, K. & Penttilä, M. The *Lipomyces starkeyi* gene *Ls120451* encodes a cellobiose transporter that enables cellobiose fermentation in *Saccharomyces cerevisiae*. *FEMS Yeast Res.* **20**, foaa019 (2020).

44. Mori, T., Kondo, O., Masuda, A., Kawagishi, H. & Hirai, H. Effect on growth, sugar consumption, and aerobic ethanol fermentation of homologous expression of the sugar transporter gene *Pshxt1* in the white rot fungus *Phanerochaete sordida* YK-624. *J. bioscience bioengineering* **128**, 537–543 (2019).

45. Delgado-Jarana, J., Moreno-Mateos, M. A. & Benítez, T. Glucose uptake in *Trichoderma harzianum*: role of *gtt1*. *Eukaryot. cell* **2**, 708–717 (2003).

46. Zhang, W. *et al.* Two major facilitator superfamily sugar transporters from *Trichoderma reesei* and their roles in induction of cellulase biosynthesis. *J. Biol. Chem.* **288**, 32861–32872 (2013).

47. Ramos, A. S. *et al.* Oxygen-and glucose-dependent expression of *Trhxt1*, a putative glucose transporter gene of *Trichoderma reesei*. *Biochemistry* **45**, 8184–8192 (2006).

48. Wang, C. *et al.* Cloning and characterization of heterologous transporters in *Saccharomyces cerevisiae* and identification of important amino acids for xylose utilization. *Metab. Eng.* **30**, 79–88 (2015).

49. Zhang, W. *et al.* Identification of residues important for substrate uptake in a glucose transporter from the filamentous fungus *Trichoderma reesei*. *Sci. Rep.* **5**, 1–10 (2015).

50. Jiang, Y. *et al.* Identification and characterization of an efficient D-xylose transporter in *Saccharomyces cerevisiae*. *J. Agric. Food Chem.* **68**, 2702–2710 (2020).

51. Casa-Villegas, M., Polaina, J. & Marín-Navarro, J. Cellobiose fermentation by *Saccharomyces cerevisiae*: Comparative analysis of intra versus extracellular sugar hydrolysis. *Process. Biochem.* **75**, 59–67 (2018).

52. Nogueira, K. M. *et al.* Characterization of a novel sugar transporter involved in sugarcane bagasse degradation in *Trichoderma reesei*. *Biotechnol. Biofuels* **11**, 84 (2018).

53. Porciuncula, J. d. O. *et al.* Identification of major facilitator transporters involved in cellulase production during lactose culture of *Trichoderma reesei* PC-3-7. *Biosci. Biotechnol. Biochem.* **77**, 1014–1022 (2013).

54. Saloheimo, A. *et al.* Xylose transport studies with xylose-utilizing *Saccharomyces cerevisiae* strains expressing heterologous and homologous permeases. *Appl. Microbiol. Biotechnol.* **74**, 1041–1052 (2007).

55. Vargas, W. A., Crutcher, F. K. & Kenerley, C. M. Functional characterization of a plant-like sucrose transporter from the beneficial fungus *Trichoderma virens*. regulation of the symbiotic association with plants by sucrose metabolism inside the fungal cells. *New Phytol.* **189**, 777–789 (2011).

56. Wahl, R., Wippel, K., Goos, S., Kämper, J. & Sauer, N. A novel high-affinity sucrose transporter is required for virulence of the plant pathogen *Ustilago maydis*. *PLoS Biol.* **8**, e1000303 (2010).

57. Wittek, A. *et al.* The fungal UmSrt1 and maize ZmSUT1 sucrose transporters battle for plant sugar resources. *J. Integr. Plant Biol.* **59**, 422–435 (2017).

58. Schuler, D. *et al.* Hxt1, a monosaccharide transporter and sensor required for virulence of the maize pathogen *Ustilago maydis*. *New Phytol.* **206**, 1086–1100 (2015).

59. Wiese, J., Kleber, R., Hampp, R. & Nehls, U. Functional characterization of the *Amanita muscaria* monosaccharide transporter, *AmMst1*. *Plant Biol.* **2**, 278–282 (2000).

60. Doehlemann, G., Molitor, F. & Hahn, M. Molecular and functional characterization of a fructose specific transporter from the gray mold fungus *Botrytis cinerea*. *Fungal Genet. Biol.* **42**, 601–610 (2005).

61. Lingner, U., Münch, S., Deising, H. B. & Sauer, N. Hexose transporters of a hemibiotrophic plant pathogen functional variations and regulatory differences at different stages of infection. *J. Biol. Chem.* **286**, 20913–20922 (2011).

62. Lingner, U., Münch, S., Sode, B., Deising, H. B. & Sauer, N. Functional characterization of a eukaryotic melibiose transporter. *Plant Physiol.* **156**, 1565–1576 (2011).

63. Voegele, R. T., Struck, C., Hahn, M. & Mendgen, K. The role of haustoria in sugar supply during infection of broad bean by the rust fungus *Uromyces fabae*. *Proc. Natl. Acad. Sci. U.S.A* **98**, 8133–8138 (2001).

64. Chang, Q. *et al.* Hexose transporter *Ps*HXT1-mediated sugar uptake is required for pathogenicity of wheat stripe rust. *Plant Biotechnol. J.* (2020).

65. Helber, N. *et al.* A versatile monosaccharide transporter that operates in the arbuscular mycorrhizal fungus *Glomus* sp is crucial for the symbiotic relationship with plants. *Plant Cell* **23**, 3812–3823 (2011).

66. Schüßler, A., Martin, H., Cohen, D., Fitz, M. & Wipf, D. Characterization of a carbohydrate transporter from symbiotic glomeromycotan fungi. *Nature* **444**, 933–936 (2006).

67. Kim, H., Lee, W.-H., Galazka, J. M., Cate, J. H. & Jin, Y.-S. Analysis of cellodextrin transporters from *Neurospora crassa* in *Saccharomyces cerevisiae* for cellobiose fermentation. *Appl. Microbiol. Biotechnol.* **98**, 1087–1094 (2014).

68. Kuorelahti, S., Kalkkinen, N., Penttilä, M., Londesborough, J. & Richard, P. Identification in the mold *Hypocrea jecorina* of the first fungal D-galacturonic acid reductase. *Biochemistry* **44**, 11234–11240 (2005).

69. Caron, L., Rousseau, F., Gagnon, É. & Isenring, P. Cloning and functional characterization of a cation-Cl^- cotransporter-interacting protein. *J. Biol. Chem.* **275**, 32027–32036 (2000).

70. Bergeron, M. J. *et al.* Frog oocytes to unveil the structure and supramolecular organization of human transport proteins. *PLoS One* **6**, e21901 (2011).

71. Wickham, H. *ggplot2: Elegant Graphics for Data Analysis* (Springer-Verlag New York, 2016).

72. R Core Team. *R: A Language and Environment for Statistical Computing*. R Foundation for Statistical Computing, Vienna, Austria (2013).

73. Boorer, K. J., Loo, D. F., Frommer, W. B. & Wright, E. M. Transport mechanism of the cloned potato H^+/sucrose cotransporter StSUT1. *J. Biol. Chem.* **271**, 25139–25144 (1996).

74. Riesmeier, J. W., Hirner, B. & Frommer, W. B. Potato sucrose transporter expression in minor veins indicates a role in phloem loading. *Plant Cell* **5**, 1591–1598 (1993).

75. Sauer, N., Friedländer, K. & Gräml-Wicke, U. Primary structure, genomic organization and heterologous expression of a glucose transporter from *Arabidopsis thaliana*. *The EMBO J.* **9**, 3045–3050 (1990).

76. Boorer, K. J., Loo, D. & Wright, E. M. Steady-state and presteady-state kinetics of the H^+/hexose cotransporter (STP1) from *Arabidopsis thaliana* expressed in *Xenopus* oocytes. *J. Biol. Chem.* **269**, 20417–20424 (1994).

77. Sauer, N. & Stolz, J. SUC1 and SUC2: two sucrose transporters from *Arabidopsis thaliana*; expression and characterization in baker's yeast and identification of the histidine-tagged protein. *Plant J.* **6**, 67–77 (1994).

78. Zhou, J.-J., Theodoulou, F., Sauer, N., Sanders, D. & Miller, A. A kinetic model with ordered cytoplasmic dissociation for SUC1, an *Arabidopsis* H^+/sucrose cotransporter expressed in *Xenopus* oocytes. *J. Membr. Biol.* **159**, 113–125 (1997).

79. Zhou, J.-J. & Miller, A. J. Comparison of the transport properties of three plant sucrose carriers expressed in *Xenopus* oocytes. *Funct. Plant Biol.* **27**, 725–732 (2000).

80. Chandran, D., Reinders, A. & Ward, J. M. Substrate specificity of the *Arabidopsis thaliana* sucrose transporter AtSUC2. *J. Biol. Chem.* **278**, 44320–44325 (2003).

81. Sauer, N., Caspari, T., Klebl, F. & Tanner, W. Functional expression of the *Chlorella* hexose transporter in *Schizosaccharomyces pombe*. *Proc. Natl. Acad. Sci. U.S.A* **87**, 7949–7952 (1990).

82. Aoshima, H., Yamada, M., Sauer, N., Komor, E. & Schobert, C. Heterologous expression of the H^+/hexose cotransporter from *Chlorella* in *Xenopus* oocytes and its characterization with respect to sugar specificity, pH and membrane potential. *J. Plant Physiol.* **141**, 293–297 (1993).

83. Gahrtz, M., Schmelzer, E., Stolz, J. & Sauer, N. Expression of the *PmSUC1* sucrose carrier gene from *Plantago major* L. is induced during seed development. *Plant J.* **9**, 93–100 (1996).

84. Gahrtz, M., Stolz, J. & Sauer, N. A phloem-specific sucrose-H^+ symporter from *Plantago major* L. supports the model of apoplastic phloem loading. *Plant J.* **6**, 697–706 (1994).
